# The Urokinase Receptor (uPAR) as a “Trojan Horse” in Targeted Cancer Therapy: Challenges and Opportunities

**DOI:** 10.3390/cancers13215376

**Published:** 2021-10-27

**Authors:** Virginia Metrangolo, Michael Ploug, Lars H. Engelholm

**Affiliations:** 1 The Finsen Laboratory, Rigshospitalet, DK-2200 Copenhagen, Denmark; virginia.metrangolo@finsenlab.dk (V.M.); m-ploug@finsenlab.dk (M.P.); 2 Biotech Research & Innovation Centre (BRIC), Department of Health and Medical Sciences, University of Copenhagen, DK-2200 Copenhagen, Denmark

**Keywords:** urokinase plasminogen activator receptor (uPAR), targeted therapy, cytotoxic approaches, translational research, theranostics

## Abstract

**Simple Summary:**

Discovered more than three decades ago, the urokinase-type plasminogen activator receptor (uPAR) has now firmly established itself as a versatile molecular target holding promise for the treatment of aggressive malignancies. The copious abundance of uPAR in virtually all human cancerous tissues versus their healthy counterparts has fostered a gradual shift in the therapeutic landscape targeting this receptor from function inhibition to cytotoxic approaches to selectively eradicate the uPAR-expressing cells by delivering a targeted cytotoxic insult. Multiple avenues are being explored in a preclinical setting, including the more innovative immune- or stroma targeting therapies. This review discusses the current state of these strategies, their potentialities, and challenges, along with future directions in the field of uPAR targeting.

**Abstract:**

One of the largest challenges to the implementation of precision oncology is identifying and validating selective tumor-driving targets to enhance the therapeutic efficacy while limiting off-target toxicity. In this context, the urokinase-type plasminogen activator receptor (uPAR) has progressively emerged as a promising therapeutic target in the management of aggressive malignancies. By focalizing the plasminogen activation cascade and subsequent extracellular proteolysis on the cell surface of migrating cells, uPAR endows malignant cells with a high proteolytic and migratory potential to dissolve the restraining extracellular matrix (ECM) barriers and metastasize to distant sites. uPAR is also assumed to choreograph multiple other neoplastic stages via a complex molecular interplay with distinct cancer-associated signaling pathways. Accordingly, high uPAR expression is observed in virtually all human cancers and is frequently associated with poor patient prognosis and survival. The promising therapeutic potential unveiled by the pleiotropic nature of this receptor has prompted the development of distinct targeted intervention strategies. The present review will focus on recently emerged cytotoxic approaches emphasizing the novel technologies and related limits hindering their application in the clinical setting. Finally, future research directions and emerging opportunities in the field of uPAR targeting are also discussed.

## 1. Introduction

Chemotherapy continues to be the first-line therapy in clinical management of patients with difficult-to-treat tumors, such as advanced metastatic diseases, where radical surgery is not an option [1]. Notwithstanding the efficacy of this conventional treatment modality, there is still an increasing demand for more targeted approaches with less adverse systemic toxicity.

Most drug development is now oriented toward the design of cancer-cell-targeted cytotoxic interventions [1]. These often consist of hybrid constructs incorporating a cytotoxic warhead linked to a tumor-targeting drug carrier (e.g., antibodies, peptides, aptamers, etc.) that provides selective drug release into and subsequent eradication of cells overexpressing the target antigen/receptor on their surface. The tumor-targeting principle could involve the cancer cells per se and/or the surrounding stromal cells in the tumor-activated microenvironment [2,3]. The improved intra-tumoral delivery bestowed by this “trojan-horse” approach confers a more favorable therapeutic profile to the cytotoxic agent by enhancing the therapeutic potential while limiting off-target toxicity. The urokinase plasminogen activator receptor (uPAR) represents one of the emerging attractive tumor targets for such targeted therapy due to its sparse expression in healthy homeostatic tissues compared to the robust expression in most solid tumors [4,5]. Internalization and recycling of uPAR could further boost the efficiency of tumor uptake of the targeted therapeutic agents [6,7,8,9]. Cytotoxic insults mediated by specific uPAR-targeting have long been underestimated in the development of new therapeutic interventions using this system. Indeed, most targeted approaches developed to date have focused on neutralizing uPAR function, primarily by interfering with its gene expression and interactions, especially with the bona fide protease ligand uPA. Although promising in some preclinical settings, none of them have advanced to the clinics so far. An in-depth overview of the mentioned approaches, along with the existing challenges hampering their clinical translation, is provided by the following comprehensive reviews [4,10,11,12,13,14,15,16,17,18,19,20,21].

Targeted cytotoxic therapy is an emerging strategy bringing a new horizon for therapeutically exploiting the urokinase receptor in cancer. It may provide valuable drug candidates that, alone or in combined intervention regimes, might lead to more robust therapeutic effects than the function inhibition approaches, which would only slow tumor growth without killing cancer cells. Distinct uPAR-binding agents, including anti-uPAR monoclonal antibodies, uPA-derived peptides, and the amino-terminal fragment of uPA (ATF, which contains the receptor-binding domain) have been widely exploited as uPAR-targeting vehicles. Similar principles have been successfully applied for the development of uPAR-directed imaging probes to detect receptor-positive malignant lesions, monitoring intratumoral drug delivery and antitumor effects of uPAR-targeted interventions, both in preclinical and clinical settings [17,22,23,24,25,26]. The design and implementation of theranostic approaches combining uPAR-targeted therapeutic and non-invasive imaging modalities, such as positron emission tomography (PET), magnetic resonance (MRI), or near-infrared (NIR) fluorescence imaging, are currently underway and hold clinical potential for significantly improving patient management and disease outcomes, as evidenced by the encouraging results achieved for prostate cancer in recent years [27,28].

After summarizing the key properties of uPAR, setting the stage for its therapeutic relevance in cancer, the present review outlines the current landscape of uPAR-targeted cytotoxic-based approaches, emphasizing the recent developments and technologies (up to July 2021). The advantages and limitations of the different strategies will be discussed, along with some of the main inherent challenges. Finally, novel opportunities and future directions of uPAR therapeutic targeting will be presented.

## 2. Biology of the Urokinase Receptor

**Figure 1:**
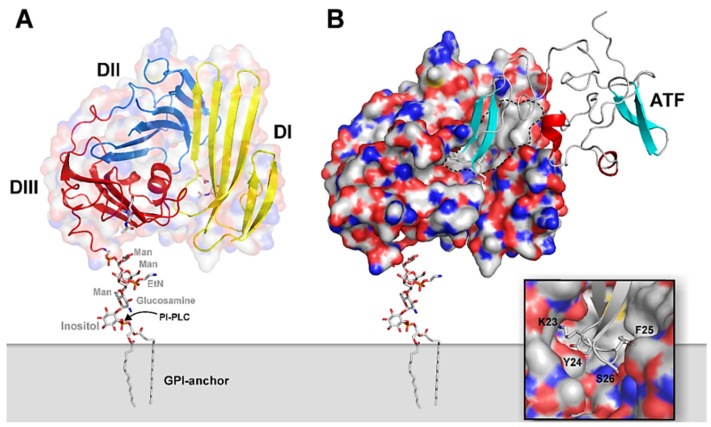
Graphical representation of the structure of uPA•uPAR complexes on the cell surface. A composite molecular model representing human uPAR based on the crystal structure solved for uPAR•ATF complexes is displayed in (**A**) using the PDB coordinates 2FD6 [36]. The molecular shape of uPAR is visualized by a semitransparent surface, while secondary structure elements are depicted as ribbons. The assembly of the three LU-domains is evident from the color coding, yellow (DI), blue (DII), and red (DIII). A hypothetical model for the GPI-anchor, tethering uPAR to the cell surface, is shown as sticks. In (**B**), the bimolecular complex of uPAR with its natural binding ligand uPA is illustrated using a solid surface representation for uPAR and a ribbon diagram for the receptor-binding fragment of uPA (ATF) used to crystallize the complex. The large hydrophobic ligand-binding cavity of uPAR is highlighted by the grey area delimited by the hatched black line using the following atomic color coding: grey (C), blue (N), red (O), and yellow (S). The inset in the bottom right corner provides a more detailed illustration of the tight engagement and burial of the tip of the β-hairpin of GFD in uPA within the deepest region of the central cavity in uPAR. Adapted from [23].

**Figure 2:**
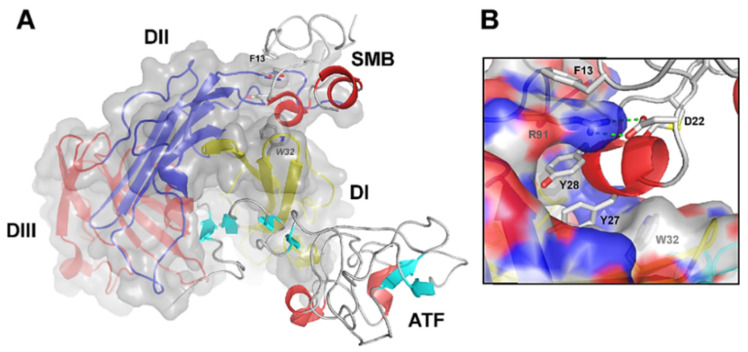
Graphical representation of the crystal structure of the ternary uPA•uPAR•Vn complex. (**A**) A composite molecular model of the ATF•uPAR•SMB complex solved by X-ray crystallography is shown in (**A**) using the PDB coordinates 3BTI [37]. The structure is rotated 90° in the horizontal axis compared to Figure 1, providing a “top view” of uPAR and moving the cell surface to the back of the picture. As in Figure 1, uPAR is represented in a composite semitransparent surface and cartoon representation. The bound ligands ATF and SMB (representing uPA and Vn, respectively) are depicted as ribbons. A detailed view of the molecular binding interface between uPAR and SMB in this ternary complex is provided in (**B**). The corresponding hot spot residues in uPAR (R91 and W32) and SMB (F13, D22, Y27, and Y28) are highlighted and shown as sticks. Adapted from [23].

**Figure 3:**
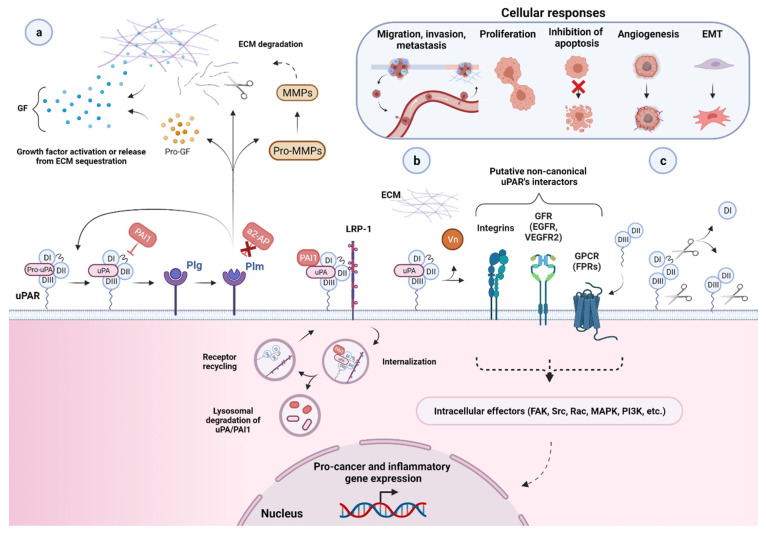
Function and regulation of the urokinase-type plasminogen activator receptor (uPAR) in the biology of cancer. (**a**) Through a high binding affinity interaction (K_D_ ~0.2 nM), the urokinase receptor binds active uPA and its zymogen pro-uPA, favoring its focused cell-surface activation. Active uPA proteolytically converts the zymogen plasminogen (plg) into active plasmin (plm), which can reciprocally activate pro-uPA, while remaining protected from its primary plasma physiological inhibitors, α2-antiplasmin (α2-AP). These mutual zymogen activation reactions start a powerful positive feedback mechanism, resulting in efficient and localized plasmin generation on the cell surface of migrating cancer cells at their leading edge. The increased cell surface concentration of the reactants involved, respectively, uPA or pro-uPA, by binding to uPAR, and plasmin and plasminogen, via multiple receptors, strongly accelerate and amplify this reciprocal activation loop. Once activated, the broad-spectrum protease plasmin mediates the non-specific proteolysis of several ECM and basement membrane (BM) components, either directly or through the activation of pro-matrix metalloproteinases (pro-MMPs), thereby promoting cancer cell migration, invasion, and metastasis. Plasmin and MMPs can also release or activate ECM-bound cancer-related growth factors (GF) contributing to tumor progression and angiogenesis. Most of these factors then feedback in an autocrine or paracrine fashion to enhance the expression of different pro-cancer genes, including urokinase plasminogen activating system (uPAS) components, such as uPA and uPAR, that further supports the proteolytic cascade and thus tumor progression. Besides α2-AP, another important physiological regulator of uPA-uPAR-induced plg-activation is the serine protease inhibitor (serpin) plasminogen activator inhibitor 1 (PAI1), which specifically inhibits uPA by forming stable ternary complexes with uPAR-bound uPA, which are subsequently internalized via the α2-macroglobulin receptor/low-density lipoprotein receptor-related protein 1 (LRP1). (**b**) By becoming a part of functional units involving distinct extracellular molecules and membrane co-receptors (e.g., its second main cognate ligand, the matrix protein Vn, members of the integrin adhesion receptor superfamily, G-protein-coupled receptors (GPCR), and growth factors receptors (GFR, e.g., EGFR and VEGFR-2, epidermal growth and vascular endothelial growth factor receptor 2)), uPAR is believed to indirectly choreograph—in a non-proteolytic fashion—several cancer-associated intracellular signal-transduction pathways regulating other tumor hallmarks, including, among others, proliferation, survival, migration, invasion, metastasis, angiogenesis, and epithelial–mesenchymal transition (EMT). The intracellular signaling components are indicated (focal adhesion kinase (FAK), Src, Rac, mitogen-activated protein kinase (MAPK), phosphatidylinositol 3-kinase (PI3K), etc.), but the pathways remain speculative. A more comprehensive list of uPAR putative non-canonical interacting proteins and related signaling consequences is reported in [4,16,73]. (**c**) Cell surface uPAR may undergo two major post-translational processing events, namely proteolytic cleavage (in the DI–DII linker region) and shedding (via hydrolysis of the GPI-anchor), resulting in diverse uPAR isoforms, including suPAR D1, suPAR DI–D3, suPAR D2–D3, and GPI-anchored uPAR D2–D3. Created with BioRender.com.

Since the initial identification, purification, and sequencing of human uPAR were accomplished around 1990 [29,30,31], a still-expanding body of literature documenting uPAR association with cancer has accumulated, and new indications continue to be uncovered. Through the combination of biochemical analysis via site-directed mutagenesis [32,33,34,35] and structural elucidation by crystallography [36,37,38,39,40,41], detailed knowledge about the uPAR structure–function relationships governing the interplay with its two cognate ligands, the serine protease uPA and the provisional matrix protein vitronectin (Vn), was outlined. As the structural and biochemical aspects of uPAR have been extensively investigated and reviewed in detail in [17,33,42,43,44], they are only briefly discussed here and summarized in Figure 1, Figure 2 and Figure 3. The pathophysiological role and expression profile of uPAR shaping its value as a cancer target will be the focus of a more detailed discussion.

### 2.1. uPAR: A Flexible Multidomain Receptor

Human uPAR is a 55–60 kDa highly glycosylated protein (283 aa residues) [45,46], tethered to the outer leaflet of the lipid bilayer of the cell membrane by a C-terminal glycosyl-phosphatidylinositol (GPI) anchor [35,47]. The extracellular ligand-binding part of uPAR consists of three homologous cysteine-rich modules (denoted DI, DII, and DIII, as numbered from the N-terminus) of approximately 90 amino acids each, connected by two flexible hinge regions [17]. These domains belong to the Ly-6/uPAR/α-neurotoxin (LU) protein domain family, which display a distinct conserved disulfide bridge pattern that creates the archetypical three-finger Ly6/uPAR (LU) domain [44,48]. Figure 1A illustrates a schematic model of the multidomain assembly of GPI- anchored uPAR, based on the crystal structure of the bimolecular complex of uPAR with the receptor binding fragment of uPA (ATF) (Figure 1B) [36]. As clearly shown, all LU-domains are intimately assembled into an almost globular structure and contribute to the formation of a central hydrophobic ligand-binding cavity, where the tip of the β-hairpin of the epidermal growth factor-like domain (GFD^1–48^) of uPA is deeply buried (as highlighted in the inset in Figure 1B). This tight interaction governs the high-affinity binding between uPA and uPAR (K_D_ ~0.2 nM), which exhibits a pronounced species-specificity between humans and mice [49,50].

This unique conformation enables the large outer receptor surface to engage in other protein interactions, e.g., Vn, as detailed in the next paragraph [38,41,51]. Vn binds uPAR at a composite epitope exposed on the DI/DII interface, via its small, N-terminal somatomedin B (SMB) domain and with a relatively weaker binding affinity (K_D_ of 2 µM) (Figure 2) [33,37,40,43]. The existence of topographically distinct uPA and Vn binding sites on uPAR enables the simultaneous binding of both ligands and, therefore, the coordinated regulation of uPAR activities (e.g., cell adhesion and pericellular proteolysis) [52,53,54].

Notably, a ~3-fold increase in the vitronectin-binding affinity is induced upon the concurrent binding of uPAR by uPA, which may act as an allosteric modulator of the vitronectin binding site, as demonstrated both biochemically, in purified systems, and in vitro [40,53,54,55].

The effective binding of uPAR to its ligands strictly depends upon the intact three-domain structure of the receptor molecule [16,43,56,57]. Indeed, proteolytic cleavage of the exposed protease-sensitive DI–DII linker region by a wide variety of proteases irreversibly impairs uPAR interactions, generating a soluble D1 fragment and a DIIDIII cleaved uPAR form, which can remain membrane-associated or shed via enzymatic hydrolysis of the GPI-anchor [5,57,58,59]. Similarly, intact uPAR can be converted to a soluble three-domain form that retains its binding capacity toward uPA and Vn (Figure 3) [5,57,58,59].

All these isoforms of uPAR generated by post-translational hydrolytic processing (suPAR DI–D3, suPAR D2–D3, suPAR D1, and GPI-anchored uPAR D2–D3) have been detected in vitro and in vivo, in both healthy and ill subjects, including cancer patients. In cancer, the plasma levels of soluble uPAR fragments are inversely associated with patient prognosis and disease outcome [4,11,16,59,60,61,62].

Circumstantial evidence from cell culture studies suggests a functional role for truncated DII-DIII uPAR forms [5,13,14,56,57,58,59,63]. Upon proteolytic cleavage, the N-terminal of the resultant C-terminal fragment may expose the chemotactic epitope sequence of uPAR located in the DI-DII linker region “^88^SRSRY^92^”, which has been shown to mimic uPA-induced directional cell migration and chemotaxis and promote angiogenesis by acting as an endogenous agonist of members of the G-protein-coupled formyl-peptide chemokine receptor family, including FPR-like 1 (FPRL1, a homolog of the formyl peptide receptor, also known as lipoxin A4 receptor, LXA4R) (Figure 3) [4,5,13,14,43,56,57,58,59,63,64,65,66,67,68,69].

These uPAR forms may therefore allegedly play a role in the process of tumor progression, possibly as autocrine or paracrine signals for tumor cell motility and angiogenesis [59,65,67].

### 2.2. Biological Functions of uPAR

From a historical perspective, uPAR was initially identified as a key regulator of extracellular-matrix (ECM) proteolysis, a fundamental process in the context of cell migration. The high-affinity binding interaction of its bona fide protease ligand uPA is, indeed, instrumental in focalizing plasminogen-activation and subsequent plasmin-mediated proteolytic activity to the cell surface of migrating cells at their leading edge, thereby greatly enhancing the efficiency of the system [70,71,72]. The biochemical aspects of this cascade have been extensively reviewed in [43] and schematically illustrated in Figure 3, which provides a graphical overview of uPAR functional involvement in cancer biology.

As elegantly shown by uPAR gene-targeted knock-out studies in mice, uPAR-mediated extracellular proteolysis is involved in several physiological and pathological tissue remodeling processes including fibrinolysis, wound healing, tissue regeneration and involution, immune response/inflammatory responses, and tumor progression in the context of malignancy (recently reviewed in [4,16]). Surprisingly, no early-onset overt phenotypic abnormalities were observed in uPAR null mice (*Plaur* ^−/−^) [74,75,76], in contrast to those genetically deficient in plasminogen (*Plg* ^−/−^) [77], implicating the existence of a functional redundancy by which other proteases, besides the physiological bona fide activators, uPA and its homolog tissue plasminogen activator (tPA), may intervene to compensate for uPAR deficiency [14,17,43,52,57,78,79]. Notwithstanding this relationship, evidence for a functional role of uPAR in plasminogen activation in vivo does exist, as shown by the severe pathogenic cutaneous alterations (pemphigoid lesions) of bitransgenic mice overexpressing uPA and uPAR under the control of the keratin 5 promoter [80]. The normal skin phenotype exhibited by either the single transgenic mice, bitransgenic mice overexpressing uPAR and a receptor-binding catalytically inactive form of uPA, or bitransgenic mice crossed into a *Plg ^−/−^* background undoubtedly underscores the strict requirement for the assembly of a functional cell surface (pro-)uPA-uPAR template to activate plasminogen [80,81]. The same prerequisite is also extended to normal physiological conditions since mice null for either Plg, uPA, or uPAR tolerate an engineered anthrax toxin, whose activity is strictly dependent on its proteolytic activation by cell-bound uPA, as detailed later [82,83].

As an obvious consequence of its role in plasminogen activation and tissue remodeling, uPAR likely facilitates cancer invasion and metastasis by providing malignant cells with a high proteolytic potential required for dissolution of ECM barriers and metastatic spread to distant sites as well as for invasive tumor growth and angiogenesis through ECM-associated GF, processed or released by plm or plm-activated MMPs (Figure 3).

Although impairment of uPAR function or inhibition of its expression was associated with impaired invasive, metastatic, and tumorigenic potential of many tumor models in vitro and in vivo [4,11,12,14,16,17,20,52,57,79,84,85], one study in transgenic mice with uPAR gene ablation showed no pronounced causal effects on disease dissemination by metastasis [86].

Besides uPAR’s well-established role in pericellular proteolysis, so-called non-proteolytic functions have emerged over the years, implying uPAR’s involvement in multiple intracellular-signaling pathways affecting several aspects of the neoplastic evolution such as cell proliferation, differentiation, survival, adhesion, migration, angiogenesis, EMT [87,88,89,90], and more [4,14,16,52,56,57,61,79,85,89]. New indications related to uPAR-function in cancer and putative signaling networks continue to be uncovered, complicating the interpretation of the functional relevance and therapeutic targeting of this receptor [91,92,93].

As a GPI-anchored receptor and, therefore, lacking an intracellular domain, signaling through uPAR is commonly believed to occur via dynamic lateral interactions in hypothetical multimolecular complexes involving diverse signaling partners and their downstream second intracellular messengers [4,14,16,44,52,56,57,79] (Figure 3). Since their discovery about 30 years ago, a wide variety of uPAR non-canonical interactors have been proposed in the literature, leading to the definition of the so-called uPAR interactome, comprising over 42 alleged protein partners so far [16,52,56,73,79,94]. In addition to the second uPAR cognate ligand, the matrix protein vitronectin, which implicates uPAR in cell adhesion and migration [43,55,63,95,96,97,98,99,100,101], at least three types of transmembrane proteins have been proposed to entertain physical contacts with uPAR and are assumed to serve as co-receptors mediating uPAR signaling in response to uPA or vitronectin. These include integrins (e.g., αMβ2 and α3β1), GPCRs, and GFR (e.g., EGFR and VEGFR-2)), with the former being the most studied and considered the most significant partners so far, as shown both in vitro and in vivo [4,14,16,43,52,56,79,102,103,104,105,106,107]. For an exhaustive overview of potential uPAR-interactors, signaling networks, and functional implications in cancer, we invite the reader to refer to the following detailed reviews [4,16,73].

Noteworthily, while the core of uPAR interactions with its key ligands, uPA and Vn, were unambiguously delineated based on crystal structure and biochemical analyses, the other claimed uPAR interplays, and functional consequences have often been inferred or indirectly demonstrated by fluorescence resonance energy transfer (FRET) microscopy, immunolocalization, or co-immunoprecipitation studies and, therefore, still remain speculative. Yet, despite the wealth of indications of direct interactions between uPAR and its putative partners (e.g., integrins), the possibility that this interaction is driven by other proteins, such as Vn or uPA, cannot be excluded, as recently demonstrated by Sidenius and colleagues [96,97,98].

More conclusive evidence along with robust structural information is essential to better discern the complex molecular networks underlying uPAR’s pathophysiological role and fully exploit its enormous potential as a cancer target.

### 2.3. uPAR Expression during Normal Physiology and in Cancer

Under normal homeostasis, baseline expression levels of uPAR are generally low or even undetectable; when present, the few sporadic uPAR-positive foci represent predominantly bone-marrow-derived blood cells, in particular neutrophils, monocytes, and macrophages, as well as quiescent endothelial cells [4,16,17,56,57,108,109,110,111]. Scattered uPAR positive cells are found in the thymus, heart, liver, spleen, lungs, and kidneys [108]. In mice, uPAR is also expressed by a subset of hematopoietic stem/progenitor cells (HSCPs) [111].

In agreement with its functional role, a remarkable up-regulation of uPAR levels transiently occurs during normal physiological processes involving cell migration and active tissue remodeling, namely embryonic development, reproduction, tissue involution, injury/wound healing, and related inflammatory/immune responses [52,57,108,112,113]. Accordingly, pathological conditions associated with chronic inflammation (e.g., central nervous and cardiovascular system disorders and immune and infectious diseases) exhibit a profound increase in uPAR expression, primarily due to infiltrating immune cells (for comprehensive reviews, the reader may refer to [114,115]). Notably, uPAR-deficient mice, although viable and fertile, exhibit functional phenotypes in congruence with these observations, such as defective leukocyte recruitment in models of bacterial infection, amelioration of inflammatory proteinuria, reduced brain damage in a model of cerebral ischemia, and depletion of a subset of HSPCs [4,16]. This implies that rather than being cell-specific, uPAR expression should be regarded as process-specific, with many cells being capable of expressing the receptor, but only during specific perturbations of homeostasis.

**Figure 4:**
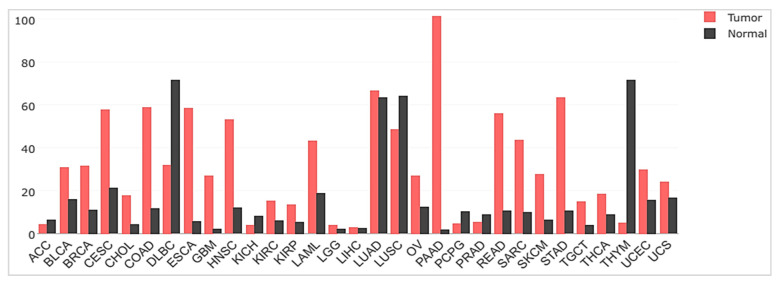
uPAR gene expression profile in human cancer. Abbreviations (TCGA, The Cancer Genome Atlas): ACC, Adenoid Cystic Carcinoma; BLCA, Bladder Urothelial Carcinoma; BRCA, Breast Invasive Carcinoma; CESC, Cervical Squamous Cell Carcinoma and Endocervical Adenocarcinoma; CHOL, Cholangiocarcinoma; COAD, Colon Adenocarcinoma; DLBC, Lymphoid Neoplasm Diffuse Large B-cell Lymphoma; ESCA, Esophageal Carcinoma; GBM, Glioblastoma Multiforme; HNSCC, Head and Neck Squamous Cell Carcinoma; KICH, Kidney Chromophobe; KIRC, Kidney Renal Clear Cell Carcinoma; KIRP, Kidney Renal Papillary Cell Carcinoma; LAML, Acute Myeloid Leukemia; LGG, Brain Lower Grade Glioma; LIHC, Liver Hepatocellular Carcinoma; LUAD, Lung Adenocarcinoma; LUSC, Lung Squamous Cell Carcinoma; OV, Ovarian Serous Cystadenocarcinoma; PAAD, Pancreatic Adenocarcinoma; PCPG, Pheochromocytoma and Paraganglioma; PRAD, Prostate Adenocarcinoma; READ, Rectum Adenocarcinoma; SARC, Sarcoma; SKCM, Skin Cutaneous Melanoma; STAD, Stomach Adenocarcinoma; TGCT, Testicular Germ Cell Tumors; THCA, Thyroid Carcinoma; THYM, Thymoma; UCES, Uterine Corpus Endometrial Carcinoma; UCS, Uterine Carcinosarcoma. Adapted from http://gepia.cancer-pku.cn/ (Accessed date: 1 August 2021).

In line with this, elevated uPAR levels, both in resected lesions or in body fluids (blood, plasma, urine, and ascites) as shed soluble suPAR (as described above and illustrated in Figure 2), have been detected in almost all human malignancies investigated to date and are frequently associated with highly invasive phenotypes, poor prognosis, and adverse clinical outcomes. The broad gene expression profile of uPAR in human cancer as reported in the Gene Expression Profiling Interactive Analysis (GEPIA) database is illustrated in Figure 4. As evident, pancreatic cancer (PAAD) is the one exhibiting the highest mean levels of uPAR mRNA, as well as the largest expression separation between neoplastic and normal tissues, compared to all other cancer types.

Not surprisingly, the adverse prognostic potential broadly associated with this receptor expression in cancer is related to its involvement in most tumor hallmarks, especially tumor invasion and metastasis. A more in-depth description of uPAR expression and its clinical diagnostic, prognostic, and predictive value in cancer can be found elsewhere [4,11,14,16,59,60,61,84,109].

Histological immunostaining and in situ hybridization studies have been instrumental in characterizing the expression profile of uPAR in human solid tumors. Although data comparison between different reports is complicated or even confounded by methodological differences (e.g., histological protocols, detection antibodies, and mRNA probes employed, lack of appropriate specificity controls, surgical specimen available, etc.) and tumor complexity, the results of most studies collectively point in the same direction and allow the identification of some general features of uPAR expression in cancer, regardless of the tumor type.

First, the expression pattern is usually complex and highly heterogeneous with varying contributions from both malignant cells and various tumor-associated cells in the surrounding reactive stroma. These typically include sub-populations of tumor-recruited inflammatory cells, mainly macrophages and neutrophils, as well as neoangiogenic endothelial cells, active fibroblasts, myofibroblasts, and rarely even neuronal cells [4,11,16,59,60,61,109]. Although the predominant cellular localization of uPAR expression appears to be tumor-specific, it is primarily confined to the stromal compartment. Pancreatic cancer is a clear example of this heterogeneity. Expression of uPAR is indeed principally confined to the desmoplastic tumor stroma, which accounts for up to 90% of the entire tumor volume [116,117,118,119,120]. There are also malignancies where the prominent uPAR expression is reported on cancer cells such as in esophageal [121,122] and cutaneous squamous cell carcinomas [40], as well as in gliomas such as anaplastic astrocytoma and glioblastomas [123,124].

Interestingly, in some cases, the tumor-specific expression patterns of uPAR seem to replicate those found in the same tissues under normal physiological remodeling and may thus underlie the functional similarities observed between normal and cancer tissue-remodeling processes (e.g., wound healing) [11,17,23,57,109,112,125,126,127].

Notably, dissimilarities in uPAR expression levels and prevalent cellular localizations are also observed among different tumor subtypes, patients with the same cancer type, and sometimes accordingly to the relative histological grade and stage with distinct impacts on the biological aggressiveness of tumors [128]. Although challenging from a therapeutical viewpoint, this substantial intratumoral heterogeneity may endorse the potential use of uPAR as a diagnostic biomarker to identify and stratify high-risk patient subgroups and therefore tailor individual-based therapies.

**Figure 5:**
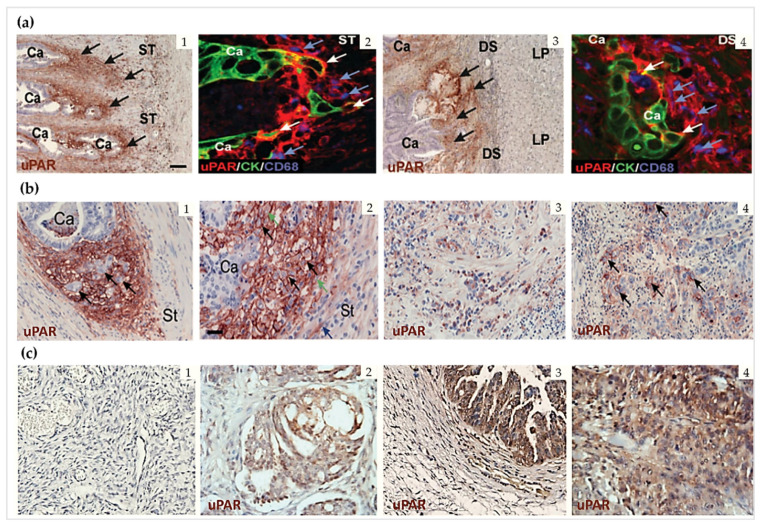
Tissue expression of uPAR in human cancer. (**a**) Peroxidase and multi-immunofluorescence staining of primary colon adenocarcinoma (panels 1–2) and a corresponding liver metastasis with desmoplastic growth pattern (panels 3–4). uPAR is primarily expressed by tumor-infiltrating macrophages (black arrows in 1 and 3; blue arrows in 2 and 4) along with some few detached budding cancer cells at the invasive cancer front (white arrows in 2 and 4) (Ca: cancer, ST: stroma, DS: desmoplastic stroma; LP: liver parenchyma). Reproduced from [129], Copyright (2009, John Wiley & Sons, Inc., Hoboken, New Jersey, USA) with permission of John Wiley & Sons, Inc. (**b**) An analogous expression pattern is seen in the intestinal subtype of gastric cancer (panels 1–2), where macrophages (green arrows in 2) and neutrophils account for the principal uPAR-expressing cells, while myofibroblasts (blue arrows in 2), at a similar location, contribute to uPAR expression to a lesser extent. In the diffuse subtype (panel 3), the uPAR-positive cells are widespread within the tumor. Despite this heterogeneity, tumor cells (black arrows) constitute a relevant fraction of the uPAR-positive cell population in both gastric cancer subtypes. Modified from [131], Copyright (2011, John Wiley & Sons, Inc.) with permission of John Wiley & Sons, Inc. (**c**) uPAR expression in normal (panel 1) and cancerous ovarian tissues grade II, III, and IV (panels 2, 3, and 4, respectively). Intense uPAR immunoreactivity is detected in both neoplastic epithelium and juxtatumoral stroma compared to the normal tissue and is significantly associated with the increasing tumor grade. Adapted from [144], Copyright (2011, American Association for Cancer Research, AACR, Philadelphia, Pennsylvania, USA), with permission of AACR.

Regardless of this complexity, uPAR overexpression is typically observed at the invasive front of the neoplastic lesion, usually at the tumor–stroma or tumor–benign tissue interface. This is particularly eye-catching at the invasive front of several types of cancers including, among others, colon [129,130], gastric [131], lower esophagus [132], hepatocellular [133,134], breast [135,136,137], prostate [138] bladder [139,140], and oral squamous cell carcinomas [141,142,143] (Figure 5).

**Figure 6:**
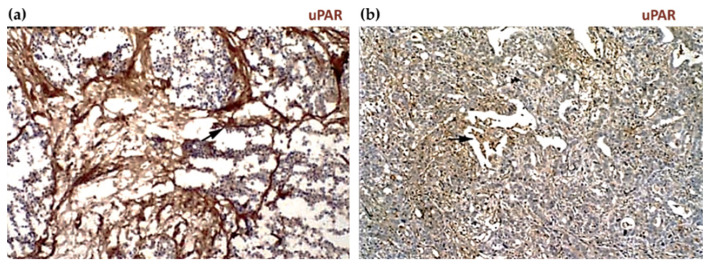
Immunoperoxidase staining of pancreatic adenocarcinomas. Metastatic pancreatic cancers show intensive staining in stromal fibroblasts (SF, **a**, black arrow), while the nonmetastatic tumors (**b**) display moderate staining in both cancer cells (black arrow) and SF. Reproduced from [145], Copyright (2007, American Association for Cancer Research, AACR), with permission from AACR.

In pancreatic cancer, uPAR immunoreactivity is consistently detected in the highly fibrotic, inflammatory or desmoplastic areas adjacent to cancer cells [120], both in the primary tumors and their paired liver metastasis [145]. In the latter, uPAR upregulation in fibroblast-like cells was found to be associated with fibrotic encapsulation of the secondary tumors, implying a functional role of fibroblasts in the metastatic process of pancreatic cancer via the uPA-uPAR pathway [145] (Figure 6).

Similarly, colon cancer liver metastasis with desmoplastic growth (Figure 5(a3)) exhibits high uPAR levels with a pattern that recapitulates the one found in the primary tumors (Figure 5(a1)) where uPAR is primarily expressed by stromal macrophages along the invasive front. On the contrary, metastasis with a pushing, or sinusoidal, growth pattern, where the neoplastic cells are in direct contact with the liver parenchyma, is essentially devoid of uPAR expression [129].

In addition to consolidating the correlate of uPAR expression with tumor progression and aggressiveness, these observations also signify the important contribution of the uPAR-positive stromal compartment to the overall process. Indeed, stromal uPAR expression may support ECM proteolysis and malignant tumor invasion and concomitantly assist the well-known tumor-promoting functions of the stroma (e.g., angiogenesis) through the receptor-mediated signaling activities [11,12,15,52,146,147]. This observation further strengthens the now well-accepted idea of cancer progression as a complex process involving a dynamic molecular interplay between malignant and supporting stromal cells that concurrently remodel the tumor microenvironment (TME) to provide sustained pro-cancer signals. Bidirectional paracrine signaling pathways intervene to regulate this complex cancer-stromal crosstalk. Soluble mediators released by cancer cells recruit and activate stromal cells such as macrophages to secrete further bioactive molecules (cytokines, growth factors, and proteolytic mediators, including uPA/uPAR), which create a permissive and supportive microenvironment for tumor growth and progression [146,147,148]. The negative prognostic value associated with uPAR stromal expression in multiple cancer types, including breast [135], colon [130], and pancreatic cancer [119], clearly emphasizes this concept and underscores the therapeutic potential of targeting the tumor stroma as a promising adjuvant anti-cancer treatment.

Distinct regulatory mechanisms control uPAR expression in tumor and associated stromal cells at multiple levels, respectively, transcriptional, post-transcriptional, and post-translational, with the former being considered the principal one [12,14,52,84,85,103,149].

uPAR transcription is driven by a variety of common cancer-associated signaling pathways via autocrine and paracrine mechanisms. Notably, the same pathways are also activated through uPAR signaling, whose overexpression may thereby establish a positive feedback loop that sustains tumor progression.

In some tumors, uPAR expression has also been detected in cancer stem cells [93,125] and bone marrow cancer cells [131,150,151]. These circulating cancer cells likely originate, at least partially, from the uPAR positive “budding” cancer cells sporadically found within the tumor stromal microenvironment such as, for example, in colon adenocarcinomas or gastric cancers, in contrast to the bulk of the primary uPAR-negative tumor [130,131], as shown by the IHC images in Figure 5a,b.

In gastric and breast cancers, uPAR expression on disseminated cancer cells in the bone marrow is an independent predictor of tumor recurrence from minimal residual disease and poor patient prognosis after surgery [151,152]. This association may have a bearing on the ability of uPAR to drive the transition between single-cell tumor dormancy and proliferation, which allows for the long-term survival of residual tumor cells during dormancy and reactivation of their proliferation years after primary treatment upon favorable conditions [13,85,91,92,153]. Simultaneous uPAR and HER2/neu gene amplification on circulating cancer cells, as well as in primary tumor cells, has also been described in advanced metastatic breast cancer (MBC) patients and similarly associated with tumor recurrence, enhanced metastatic potential, and unfavorable outcomes [154]. Such co-amplification suggests the proposed HER2–uPAR cooperativity as being specific to an early-stage aggressive breast carcinoma subtype and may partially explain the failure of existing HER2-targeted therapies and drug resistance, thereby indicating these receptors as potential synergistic targets for therapeutic intervention [154]. Besides breast cancer, uPAR amplification has also been frequently reported in pancreatic cancer and analogously recognized as a significant adverse prognostic parameter identifying a subgroup of particularly aggressive tumors [10].

This brief overview of uPAR expression provides a glimpse into the existing clinical evidence advancing uPAR as a prognostic cancer biomarker and a possible therapeutic target in several common malignancies, as specified in the following section.

## 3. uPAR: A Potential “Gateway” for Cytotoxic Cancer Therapy

The pathophysiological role and expression of uPAR in most aggressive cancer lesions, the related prognostic value in many of them, coupled to the apparent lack of overt phenotypes associated with uPAR deficiency, all highlight uPAR as a potential candidate in targeted cancer therapy.

Most experimental strategies explored to date have focused on restraining pericellular uPAR-mediated plasminogen activation, mostly by interfering with the receptor gene expression and interaction with its bona fine ligand, uPA. These include monoclonal antibodies, small molecules- and peptide-derived antagonists (recently reviewed in [62]), recombinant uPA-derived fusion proteins, and various gene therapy approaches. However, although promising in a preclinical setting, none of them have advanced into clinical evaluation. Species-specificity, tumor model limitations, and a rapidly evolving landscape on the relevant determinants and functions of uPAR to target are the main hurdles to the development of uPAR antagonists. The later evidence of uPAR putative involvement in signaling cross-talks with other cancer-associated protein partners has provided an alternative, yet challenging, opportunity to explore therapeutically targeting these interactions and potentially interfere with uPAR functions downstream of uPA proteolytic activity. However, the potential of this approach remains an open question, and future studies elucidating the current controversies underlying uPAR signaling, particularly within in vivo models, will help the field advance.

An in-depth overview of the mentioned approaches, along with the existing challenges hampering their advance into the clinics, is provided by the following detailed reviews [4,10,11,12,13,14,15,16,17,18,19,20,21,43].

The last decade has brought new avenues in cancer treatment focusing on targeted cytotoxic therapies [1]. The widespread overexpression of uPAR in most malignant tissues as compared to their normal counterparts renders uPAR a selective and versatile tool for delivering a direct cytotoxic insult to uPAR expressing cells, leading to their targeted eradication.

This targeting strategy steadily gains momentum and is showing promise in preclinical studies. Different avenues have been and are currently being explored, including immunotherapy approaches. The rationale behind them, related advantages and drawbacks, will be discussed in-depth in the following sections.

In most cases, uPAR targeting is accomplished using monoclonal antibodies, uPA-derived peptides, and a high-affinity receptor-binding fragment of uPA (ATF_1-135_, which contains the GFD). These ligands provide effective binding scaffolds for conjugation and targeted delivery of different types of cytotoxic payloads or effectors, including traditional anticancer agents, cytotoxic products, radioisotopes, photosensitizers, chimeric antigen receptor (CAR) T-cells, oncolytic virus, or even immunostimulators.

Not only does this approach enhance tumor-specificity, but it also improves intratumoral delivery as uPAR-dependent internalization provides a gateway for targeted intracellular drug release, thereby optimizing the therapeutic response while reducing systemic toxicity. Given the intratumoral heterogeneity in uPAR expression, it is important to characterize this expression pattern when designing an optimal uPAR-targeted cytotoxic insult, as it would strongly impact its effectiveness. The remarkable stromal expression of uPAR also allows targeting this compartment, which offers multiple opportunities to enhance the therapeutic efficacy compared to exclusively targeting the tumor cells, especially in tumors expressing uPAR in both cell types, such as pancreatic cancer, or those lacking a tumor-specific molecular target [11,12,15,52,120,146]. Indeed, while having an indirect anti-cancer effect by attenuating the tumor-promoting effect of the stroma, cytotoxic targeting of this compartment may also increase drug-delivery efficiency by breaking down the dense stromal barrier, which severely hampers tumor perfusion by therapeutic agents, ultimately leading to drug resistance and disease recurrence [3].

In principle, as the tumor stroma predominantly accounts for uPAR expression in most cancer types and patient subgroups, effective tumor regression and/or eradication would ideally be achieved by implementing combined strategies with cytotoxins targeting both the cancer cells per se and the surrounding activated tumor stroma. An intriguing option that is gaining growing interest involves the use of stromal cells, especially tumor-associated macrophages (TAMs) [155,156], as potential autologous delivery vehicles for a localized tumor bystander effect that would indirectly enhance the killing of neighboring cancer cells with low or no uPAR expression. Alternatively, immunosuppressive uPAR-positive TAMs may also be reprogrammed to restore their immunostimulatory/tumoricidal properties and possibly potentiate immune checkpoint blockade therapies (anti-PD-1/PD-L1/CTLA-4 antibodies), as already observed for other therapeutic targets [155,156]. Although still in its infancy, uPAR-mediated stromal targeting is now becoming an attractive avenue, holding promise for the design of combinatorial intervention strategies that may benefit future cancer treatment.

It is also relevant to mention that although uPAR expression levels are generally low in most vital tissues, the baseline expression in the glomeruli of normal kidneys raises concerns about potential cytotoxic implications of such treatment modalities, as will be discussed later.

### 3.1. uPAR-Targeted Radionuclide Therapy

Radiopharmaceutical therapy (RPT) is rapidly emerging as an effective and safe targeted approach to treating various cancer types, with radiolabeled peptides and antibodies representing important targeting vehicles [157,158]. In this context, the most successful example, so far, regards somatostatin-based targeting of neuroendocrine tumors (e.g., ^177^Lu-DOTA-TATE, ^90^YDOTA-TOC) [159]. Among the benefits of this approach is the possibility to target disseminated cancer cells that have spread throughout the body and cause tumor relapse, limiting the efficacy of conventional radiotherapy.

Although still at its early stage, a few uPAR-targeted radiopharmaceuticals have been constructed in the last decade (Table 1). As for most RPTs, these agents are re-engineered versions of existing compounds used for nuclear imaging. As alluded to in the previous sections, the ubiquitous uPAR expression at the tumor–stromal interface of several invading cancer lesions makes it an appealing molecular imaging target for the clinical assessment of tumor invasion and metastatic dissemination. Accordingly, a plethora of uPAR-targeting PET-probes mostly based on the high-affinity 9-mer antagonist peptide AE105 have been synthesized and tested preclinically in diverse human xenograft mouse models and, recently, also in two clinical uPAR PET studies in humans, including prostate, breast, and bladder cancer patients, with promising results [17,22,23,24,25,28,160]. A thorough overview of the versatile applications of AE105 for non-invasive imaging of cancer is provided by the following review [26].

In preclinical studies, AE105 was explored for targeted delivery of highly ionizing α- or β-emitting nuclides for therapeutic intervention, thus setting the very early stage for a uPAR-targeted therapy. The dual use of AE105 as an imaging and therapeutic agent (theranostics) provides an additional opportunity as a companion diagnostic to optimize cancer management by stratifying patients that may benefit from the treatment.

An inherent limitation in the preclinical use of AE105 is its strict species-specificity (does not bind mouse uPAR), which complicates the evaluation of undesired toxic side effects such as nephrotoxicity in mouse models using human tumor xenografts [161]. This aspect is even more relevant when targeting uPAR, whose baseline expression in the kidneys, and other non-target organs, such as the bone marrow, may further exacerbate the potential PPRT-induced damage to these organs.

Sebastian et al., published the first report of a uPAR-targeted radiopharmaceutical. They synthesized a ^213^Bi-labeled-DOTA-conjugated dimer of AE105, designated ^213^Bi-P-P4D, for α-emitter radiotherapy of uPAR-positive advanced ovarian carcinomas in cell culture [162]. ^213^Bi-P-P4D bound specifically to uPAR-overexpressing human monocytic U937 and OV-MZ-6 ovarian cancer cells, as demonstrated by competitive binding studies using pro-uPA, or the soluble receptor form, suPAR. A clear dose-dependent correlation between the OV-MZ-6 survival rate and ^213^Bi-P-P4D activity was established by a colony-forming assay [162]. Biodistribution of ^213^BiP-P4D was studied in nude mice bearing intraperitoneal (i.p.) OV-MZ-6 cell-derived tumor nodules, following i.p. injection. ^213^Bi-P-P4D uptake by tumor tissue was higher than in all other organs throughout the observation period, except for pancreas and kidney, but overall within the range of other published radiolabeled peptides in tumor-bearing mice [148]. Kidney uptake was distinctly reduced (~50%) following the pre-administration of the plasma expander gelofusine, which is among the compounds currently investigated to prevent or limit renal damage by radiopeptides, both in preclinical and clinical settings [163,164]. Unfortunately, the specificity of ^213^Bi-P-P4D tumor accumulation was not substantiated by proper controls, and the in vivo therapeutic efficacy was not evaluated, thus demanding a further preclinical investigation.

Persson et al. provided the first proof-of-concept evidence of localized radiotherapy using a DOTA-AE105 derivative radiolabeled with the therapeutic β-emitter ^177^Lu in a human colorectal HT-29 xenograft cancer model [165]. The treatment (two doses administered on days 0 and 7, respectively) induced a significant targeted effect on both tumor size and the number of uPAR positive cancer cells compared to non-binding analog (^177^Lu-DOTA-AE105mut) and vehicle control-treated groups [165]. Although all uPAR-expressing cells were eradicated by this treatment, the reduction of tumor size was only moderate and transient due to the combined small penetration range of ^177^Lu β-radiation (<2 mm) and the confined expression of uPAR only at the periphery of the tumor comprising only 10% of the total HT-29 cancer cells in the tumor lesion [165]. As in the former report, the kidneys had the highest accumulation of radioactivity as they constitute the secretion pathway of AE105—this effect is not caused by tracer binding to murine uPAR-positive podocytes due to AE105 species-specificity. No gross histopathological changes were observed on H&E-stained kidney sections nor weight differences, though this does not preclude that toxicity may occur if the host-derived uPAR were to bind AE105 [165].

^177^Lu-DOTA-AE105 was also well-tolerated in a mouse model of human disseminated prostate cancer, where it demonstrated a pronounced anti-metastatic effect leading to a significant reduction in metastatic foci and prolonged metastatic-free survival versus the control groups [165]. In both models, the uPAR-positive primary and metastatic lesions were accurately detected by PET imaging using the above-mentioned uPAR-imaging probe, ^64^Cu-labeled DOTA-AE105. Despite providing in vivo evidence of a uPAR-specific targeting effect, the full theranostic potential of this targeted radiotherapy approach, and related systemic adverse toxicity (e.g., nephrotoxicity), still needs further evaluation due to the species-selectivity inherent to AE105. The full cytotoxic effect on human tumor lesions in xenograft mouse models is probably underestimated as it leaves the host stromal compartment essentially unharmed, as stated before [161].

The same consideration also applies to another study that similarly explored the theranostic targeting of uPAR in a metastatic breast cancer model using a ^111^In/^177^Lu-conjugated recombinant anti-uPAR antagonist antibody 2G10 IgG [166,167]. This antibody, identified from a human phage display library, binds human (K_D_ ~10–40 nM) but not mouse uPAR, prevented the uPA-uPAR interaction, and showed in vivo diagnostic and therapeutic efficacy in triple-negative breast cancer (TNBC) tumor xenografts and a metastatic mouse model [166,167]. The radioimmunotherapy (RIT) study performed with ^177^Lu-2G10 IgG resulted in complete tumor regression. However, the intrinsic antitumor activity exhibited by this antibody in its unlabeled form complicates the definition of the added beneficial theranostic impact deriving from the radionuclide targeting per se.

Although further preclinical validation is required, and notwithstanding the existing limitations in toxicity assessment, the availability of human anti-uPAR targeting vehicles and their successful application as imaging agents firmly support the clinical translation of the present findings to determine the theranostic utility of uPAR in the management of aggressive tumors, such as prostate or breast cancers.

**Table 1:** uPAR-targeted radiotherapy interventions.

Compound	Sequence/Structure of the uPAR-Targeting Moiety	Radionuclide	Application	Model System	Ref.
[Bi^3+^]-DOTA–(linker^4^-AE105)_2_	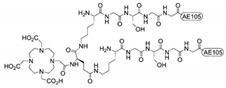	^213^Bi	Preclinical	Ovarian cancer cells and related xenograft mouse models	[162]
[Lu^2+^]-DOTA-AE105	^ 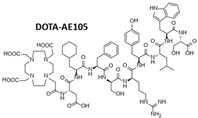 ^	^177^Lu	Preclinical	Human prostate and colorectal cancer cells and relative xenograft mouse models	[165]
[Lu^2+^]-2G10	Recombinant human anti-uPAR IgG identified from a human phage display library	^177^Lu	Preclinical	Triple negative breast cancer cells and relative xenograft mouse models	[166]

### 3.2. uPAR-Targeted Recombinant Fusion Toxins, Protease-Activated Prodrugs, and Antibody-Drug Conjugates

#### 3.2.1. uPAR-Targeted Recombinant Fusion Toxins

Another class of targeted cytotoxic approaches is represented by recombinant immuno- and ligand-targeted toxins (IT and LT) [168,169,170,171,172]. In these fusion constructs, the tumor-targeting moiety—the receptor-binding antibody/antibody fragment or endogenous ligand—is armed with the catalytic domain of highly potent cytotoxic products, usually microbial or plant protein toxins, to achieve selective tumor potency and thus the killing of designated target cells [168,170,171]. Indeed, the construct is devised to replace the cell-binding domain of the toxin with the tumor-targeting vehicle to dictate the desired binding specificity [168]. Toxin-induced cell death generally occurs via apoptosis through irreversible inhibition of protein synthesis after internalization and intracellular processing of the construct [168,170,171]. Because of the catalytic potency of the toxin moiety, a small number of molecules delivered to the cytosol may kill the target cells [173]. Unlike radioisotope immunoconjugates, the internalization of the construct is strictly required for the toxin to exert its cytotoxic action on the respective intracellular targets. Various toxins have been used for this purpose, but the most intensively employed include mutated *Pseudomonas aeruginosa* exotoxin A (PE) and Diphtheria toxin (DT), along with plant toxins, such as ricin and saporin [168,170,171,174]. Genetic engineering of the toxin moiety has been pivotal for eliminating two of the main hurdles related to this class of therapeutics, namely too low toxicity and immunogenicity [169]. Nevertheless, their production has increasingly gained interest in the biopharma sector, as evidenced by the growing number of recombinant chimeras currently undergoing preclinical and clinical investigation for solid and, especially, hematological tumors with promising results. The most successful examples are Denileukin diftitox (Ontak) and Tagraxofusp (Elzonris), two DT-derived chimeras targeting the IL-2 and IL-3 receptors, approved by the FDA for the treatment of cutaneous T-cell lymphoma and blastic plasmacytoid dendritic cell neoplasm, respectively [168,170,171]. Despite its promise, Denileukin diftitox has been clinically discontinued due to production issues inherent to the bacterial recombinant expression system used, *E. coli* [175], the choice of which is critical in the development process of recombinant therapeutics, as described later. Recently, a PE-based immunotoxin targeting CD22 has been approved for therapy of hairy cell leukemia [169].

Various uPAR-targeted recombinant fusion toxins have been developed using the high-affinity catalytically inactive ATF_1-135_ of human uPA (containing the receptor binding domain, GFD) [176] as the molecular entity enabling the specific targeting of uPAR positive cancer cells [177]. The various cytotoxic warheads were mostly derived from DT, PE, and saporin toxins.

Several DT-based recombinant fusion proteins have been developed to target uPAR-overexpressing GBMs, where targeted-toxin therapy has been successfully applied, holding promise as a new adjuvant treatment for the therapy-resistant forms [178]. Despite its highly invasive nature, GBM rarely metastasizes outside the brain, allowing for targeted agents to be directly delivered to the tumor, thus avoiding toxicity issues associated with systemic delivery. Besides uPAR, the most targeted GMB surface antigens include the aberrantly overexpressed IL-13, IL-4, and EGF receptors. However, due to the inconsistent receptor expression in these tumors, no single fusion protein would ideally be inclusive in recognizing all the different GBM forms [178]. DTAT, the first monospecific uPAR-targeted DT-fusion protein, was designed by Vallera et al. to address this question. It was conceived as an alternative strategy for GBMs unresponsive to treatment with DTIL13, another fusion protein targeting the IL-13 receptor [179]. DTAT consists of the ATF of uPA fused with the first 388 amino acid residues of DT encoding the catalytic and translocation domains (DT388). Choosing uPAR to target both GBM cancer cells and the associated uPAR-positive tumor neovasculature seemed advantageous, potentially enhancing the therapeutic efficacy by depriving the growing tumor of its essential nutrient supplying source [180]. Based on DTAT, bispecific ligand-directed toxins (BLT) were subsequently developed to improve the efficacy of anti-GBM therapeutics while reducing the cost associated with combination therapies [174]. The first example, DTAT13, was constructed by coupling ATF to DTIL13 to retain site-specific binding to both uPA and IL-13Rα2 receptors and further broadening the reactivity against GBM [181]. In vitro, both glioblastoma cells and human umbilical vein endothelial cells (HUVECs) were selectively killed by DTAT and DTAT13 in a dose-dependent manner with nanomolar IC_50_ values [182]. However, due to differential receptor expression, neither agent was able to eradicate all GBM cell lines tested, thus confirming the previous assumption that no single targeted toxin is likely to be universally effective against heterogeneous tumors such as GBM. On the other hand, the requirement of both uPA and IL-13 receptors for DTAT13 cytotoxicity was shown by the lack of sensitivity in receptor-deficient cells and the inhibitory effects of receptor-blocking antibodies [181,182]. In vivo, both DTAT and DTAT13 induced rapid regression of subcutaneous xenotransplanted human GBM tumors when administered either subcutaneously or intracranially. However, DTAT13 appeared to be more effective than DTAT in U87MG tumor-bearing mice, whose tumor relapse was delayed following DTAT13 treatment. In contrast, in U373 tumors, DTAT13 had similar inhibitory effects to DTIL13, as observed in vitro, thus confirming the impact of receptor expression heterogeneity on drug efficacy [181,182]. Additionally, DTAT13 was 160- and 8-fold less toxic than DTAT and DTIL13, respectively, probably due to the favorable pharmacokinetic properties conferred by its larger size compared to small conjugates, as reasoned by the authors [179,181,182,183]. Pharmacokinetic studies showed that DTAT13 exhibits properties of both the native fusion proteins, which endows it with an improved therapeutic profile [183].

DTAT was further tested in an intracranial xenograft model that more accurately mimics the clinical use of this agent, namely intracranial delivery, compared to subcutaneous tumor models [184]. Administration of the fusion toxin to the brain resulted in a significant decrease in tumor volume and doubled the survival time, an impressive result since mice were treated when the tumor burden was advanced, more closely mimicking a clinical course for GBM [184]. Magnetic resonance imaging (MRI) was successfully employed to monitor tumor growth, treatment toxicity, and efficacy, demonstrating the utility of this imaging tool in brain tumors [184]. Together with IHC analysis, MRI confirmed no DTAT-related vascular leak syndrome, which is one of the major dose-limiting toxicity determinants associated with the use of IT/LT that may preclude their clinical use [169,170,171,185]. Yet, consistent with other published reports, a 16-fold increase in the maximum tolerated dose (MTD) was observed following stereotactic administration of DTAT at the tumor site via convection-enhanced delivery (CED), which is typically employed in brain tumor clinical trials [184,186].

A dual-targeting DTAT-based BLT (DTATEGF) was synthesized to simultaneously target uPAR- and EGDR-expressing non-small-cell lung cancer (NSCLC) that has metastasized to the brain [187]. In vivo, a xenograft intracranial tumor model was established using human NSCLC cells transfected with a firefly luciferase reporter gene (NSCLC-luc) to monitor intracranial tumor growth using real-time bioluminescent imaging (BLI). Compared to the parental monospecific cytotoxins, DTATEGF exhibited more than 100- to 1000-fold more potent antitumor efficacy both in vitro (IC_50_ = 0.001 nM) and in vivo when administered intracranially by CED via an osmotic minipump, providing a significant survival benefit relative to the controls [187].

Overall, these encouraging preclinical results underscore the potential of bispecific agents, such as DTATEGF and DTAT13, whose enhanced targeting specificity may improve treatment of heterogeneous tumors while simultaneously reducing off-target toxicity, thereby warranting their further clinical investigation [181]. Importantly, the use of xenograft models employed in these studies, although informative, impeded the evaluation of the potential uPAR-induced vascular effect of the DT-chimeras, and related systemic toxicity on normal organs, due to the species-specificity barrier posed by the ATF moiety, as already observed for AE105 or 2G10 Ab [49]. However, the fact that pigs, which express cross-reactive uPAR and whose brain size is about a tenth of a human brain, tolerated 2 µg of intracranially administered DTAT without evidence of toxicity indicates that a therapeutic index may exist, mandating further studies [184]. A second limitation of these studies is that they did not address the potential immunogenicity of the DT-targeted toxins, which is one of the principal drawbacks affecting the clinical application of this class of therapeutics, as patients with neutralizing antitoxin antibodies, which frequently appear after one or two treatment cycles, rapidly clear the fusion proteins from the bloodstream dramatically compromising their efficacy [169,170,177]. This phenomenon is even more relevant for DT-based toxins due to DT preimmunization in most people, which leads to a pretreatment blood antibody titer in many patients and subsequent mounting of anamnestic immune responses after DT conjugate therapies such as, for example, Denileukin diftitox [169,170,177].

Different strategies have been devised to address this issue, such as the construction of toxin variants with diminished antigenicity by mutagenic deletion of immunogenic epitopes [169,170,177,188,189]. In the context of uPAR targeting, an example is provided by another BLT that, similarly to DTAT and DTAT derivatives, was designed for simultaneous targeting of EGFR overexpressing GBMs and uPAR on the associated neovasculature [190,191,192]. In this case, the targeting ligands (respectively, human EGF and ATF) were spliced to a truncated variant of *Pseudomonas* exotoxin A (PE38) modified with a C-terminal Lys-Asp-Glu-Leu (KDEL) sequence, which increases the toxin potency by enhancing its retention in the endoplasmic reticulum (ER) [190,191,192]. Further, critical amino acids in each of the seven mapped immunodominant epitopes in PE38 were mutated to reduce the toxin immunogenicity without a loss of catalytic activity [193]. This toxin, originally called EGFATFKDEL-7mut, became later known as EGFR-targeted bispecific angiotoxin (eBAT) [190,191,192]. In vitro, eBAT was selectively active in the picomolar range against both human glioblastoma cell lines and HUVECs cells, thus proving its antiangiogenic potential [191,192]. PE38 with reduced antigenicity did not reduce drug activity compared to the nonmutated parental form. In vivo, aggressive brain tumors were grafted subcutaneously and intracranially in athymic nude mice and rats, respectively, using human U87GM-luc glioma cells and then monitored by real-time BLI to assess the therapeutic response. A novel hollow fiber catheter system was employed in rats for intracranial delivery of eBAT via CED to improve drug distribution while reducing backflow compared with conventional single-port catheters [190,192]. Treatment significantly reduced tumor growth of the established small gliomas in two independent experiments and resulted in some long-term disease-free survivors (>130 days post-tumor inoculation), without apparent treatment-related systemic toxicity [190,191,192]. As already seen for the above-described uPAR-targeted BLT, DTAT13 and DTATEGF, eBAT was consistently more effective than its monospecific counterparts, ATFKDEL and EGFKDEL, or an equimolar combination of the two, both in vitro and in vivo [190,191,192]. Remarkably, the adaptive immune response toward PE was successfully abated (<90%), underscoring the relevance of toxin deimmunization strategies to mitigate immunogenicity risks in potential future trials involving IT/LT [172]. Indeed, humans recognize the same PE epitopes as mice, supporting the latter’s utility as a model for human immunogenicity [194]. Unfortunately, in vivo evidence of eBAT uPAR-mediated vascular effects could not be provided due to the use of xenograft models, similarly to the previously reported studies. Hence, to investigate this aspect and enable better interpretation and potential translation of the preclinical findings, adequate model systems should be employed such as knock-in humanized models expressing human uPAR or, more simply, xenograft murine cancer models. The latter will require the use of murine ATF as a targeting moiety.

eBAT effectiveness was further proven against other cancer cell types and related in vivo xenograft models, respectively, human head and neck squamous cell carcinomas, breast cancer [195], and pediatric sarcomas [196]. As observed in GBM, eBAT was more potent than its monospecific form. Accordingly, cancer cells expressing both receptors were more sensitive to eBAT than the monospecific targeted cells and related in vivo models [196]. Although the differential sensitivity of cell lines to the toxin may play a role, the increased potency may likely result from the BLTs’ superior binding affinity (due to avidity effects) to dual-receptor expressing cells, EGFR- and uPAR-expressing cells in the case of eBAT, as confirmed by flow-cytometry analysis in GBM cells and, recently, in other sarcoma and ovarian cancer cell lines. Noteworthily, in the latter, a more pronounced difference was observed in the IC_50_ between eBAT and ATFKDEL than that reported between eBAT and EGFKDEL, probably due to the relatively low expression of uPAR on these cells as compared with EGFR, which was the primary determinant of cytotoxicity [197]. Thus, bispecific targeted agents, such as eBAT, may also potentially overcome deficiencies in individual receptor expression while retaining high potency [195]. In vivo, eBAT was well-tolerated, and no adverse effects arose at the concentrations used based on the unchanging average mouse weights [195,196]. Yet, cutaneous, ocular, and gastrointestinal dose-limiting toxicities, typically associated with EGFR-targeted therapy in humans [198], were not reported. This was likely due to the cytotoxic activity of eBAT that does not interfere with EGFR signaling per se, along with the enhanced targeting specificity conferred by its bispecific nature.

A recent study of canine hemangiosarcoma (HSA) validated this line of argumentation. HSA is an aggressive and incurable spontaneous tumor whose histopathology resembles human angiosarcoma (AS), thus offering a valuable comparative model for this and potentially other aggressive cancer types [199]. Indeed, as confirmed by HIC analysis, the expression pattern of receptors, including EGFR and uPAR, is almost overlapping between HSA and AS, allowing for non-species cross-reactive agents, such as eBAT, to be tested in “on target models” and, therefore, in a more clinically translatable setting compared to “nontarget” xenograft models.

In vitro, eBAT effectively killed both human HA and canine HSA cells as well as canine hemangiospheres enriched with cancer stem cells at clinically relevant subnanomolar doses [199]. Encouraged by this result, an adaptative dose-finding, Phase I–II clinical trial—the first ever performed—was undertaken in which 23 dogs with spontaneous low-stage HSA received one cycle of intravenous eBAT after splenectomy and before doxorubicin chemotherapy. eBAT was well-tolerated at the biologically effective concentration of 50 µg/kg, with none of the dogs experiencing common EGFR-targeting associated toxicities or signs of capillary leak syndrome [199]. Reversible liver toxicity was only observed in two cases. Impressively, eBAT almost doubled the 6-month survival rate (~70%) of dogs receiving the biologically active dose versus the comparison group treated with standard of care alone (<40%). Six dogs were long-term survivors, living over 450 days [199]. Anti-eBAT antibody responses were observed in less than half of the dog population without interfering with eBAT efficacy, as already seen in GMB. Collectively, these studies provide a strong rationale for clinical translation of this BLT as adjuvant therapy to supplement standard of care therapies for treating both residual and metastatic, relapsed cancers, such as sarcomas, and potentially other EGFR- and uPAR-expressing cancers. Further testing, along with pharmacokinetic studies, will be instrumental for elucidating the exact mechanisms underlying the in vivo efficacy and excellent safety profile of this bispecific ligand-directed toxin and optimize the appropriate treatment strategy for future clinical trials in humans, considering the existing relevant species-related physiological differences.

Another example of a monospecific uPAR-targeted fusion toxin is ATF-SAP, comprising the plant-derived type I ribosome inactivating protein (RIP) saporin (SAP), isolated from the seeds of *Saponaria officinalis* [200]. The lack of a receptor-binding domain in these types of RIPs makes them excellent candidates as catalytic moieties to produce such recombinant chimeras [201]. Among Type I RIPs, SAP has been extensively employed due to its potent enzymatic activity and thermal and proteolytic stability, as well as better tolerability and reduced immunogenicity compared to bacterial toxins, rendering it a valuable tool for cancer therapy [201]. Its internalization mechanism, which remains still controversial for most of Type I RIPs, has been demonstrated to involve the low-density lipoprotein receptors (LDLR) family and, in particular, LRP1 [201,202], which is also known to mediate the physiological internalization of receptor-bound urokinase/PAI-1 complexes [6,7]. Elucidating the intracellular routing of such targeted toxins is fundamental for their optimal use as anticancer drugs since their biochemical targets reside in the cytoplasm.

The production of ATF-SAP, initially based on the traditional *E. coli* expression system [203,204], was later set up in *Pichia pastoris* GS115 yeast [205] due to the misfolding and degradation of the structurally complex ATF moiety in the bacterial host cells, resulting in low product yields, as well as the advantageous presence of a characteristic eukaryotic secretory pathway. ATF-SAP was efficiently and safely expressed in the GS115 yeast strain, following codon usage optimization of the fusion construct [205,206]. A fermentation process with lab-scale stirred bioreactors was successfully implemented to produce discrete homogeneous batches of ATF-SAP. In vitro, selective cytotoxicity was demonstrated against uPAR-overexpressing U937 leukemia cells with a IC_50_ of 0.1 nM [206]. Recently, the therapeutic potential of ATF-SAP has also been investigated in other putative candidate uPAR-overexpressing tumor entities, including breast and bladder cancers [207]. ATF-SAP selectively killed the in vitro models in a dose-dependent manner and proportionally to uPAR surface density, with IC_50_ values in the nanomolar range, as found for the U937 cells [207]. In both cases, these values were 100 times lower compared to that of untargeted SAP, further evidence of the targeting efficiency of the ATF moiety that enhances SAP delivery to the receptor-positive target cells [207]. However, an absolute correlation between uPAR expression and ATF-SAP cytotoxicity was not observed, as proven by the unexpected lack of activity of ATF-SAP toward highly uPAR expressing cells such as fibroblasts and the triple-negative breast cancer cell line, MDA-MB-231. The authors attributed this phenomenon to potential differences in the internalization process of the ATF chimera in distinct cell lines based on the relative differential expression of uPAR and accessory molecules involved in the receptor internalization, uPA, PAI-1, and LRP-1, which has also proven to be involved in ATF-SAP receptor-mediated endocytosis [203,204]. Although potential uptake mechanisms have been postulated by the authors, further study is needed to appropriately address this aspect, as no internalization assays were performed, and the assumptions were purely based on the expression data of the investigated internalizing components. Indeed, in addition to the most characterized ligand-induced LRP-1-mediated route, other endocytic pathways may regulate uPAR intracellular trafficking, including a ligand- and LRP1-independent macropynocitic-like mechanism identified by Cortese et al. as responsible for uPAR constitutive endocytosis and recycling [8].

Intravenous administration of ATF-SAP in an allogenic xenograft mouse model of bladder cancer significantly delayed tumor growth and increased animal overall survival, without eliciting noticeable toxic effects such as weight loss or lethargy [207]. However, immunogenicity was not evaluated in this study, thus demanding a future investigation. Overall, the present studies, along with the availability of a fermentation strategy for large-scale production, signifies the promising therapeutic potential of this toxin chimera that encourages its further preclinical evaluation. The main features of the herein described ATF-toxin fusion proteins are summarized in Table 2.

#### 3.2.2. uPA-Activated Prodrugs

Besides bispecific targeting, another exciting approach to finely improve the therapeutic profile of fusion toxins implies the construction of tumor-selective prodrugs that are site-specifically activated at the intended tumor site by tumor overexpressed pericellular proteases [172,208]. Bacterial toxins such as DT, PE, and anthrax toxin are particularly amenable to this strategy as their cytotoxic activity is proteolytically unleashed on the cell surface in an early step of the intoxication process [172]. This strict and unique requirement enables the toxins to be engineered to make their activation depend on a tumor-associated protease and, accordingly, endows the resulting recombinant construct with the ability to selectively act on target protease-overexpressing cancer cells while sparing the healthy counterpart. Not surprisingly, the broad and localized activity of uPA in most tumors has provided an excellent opportunity for implementing such a strategy [11,14,17,209].

**Figure 7:**
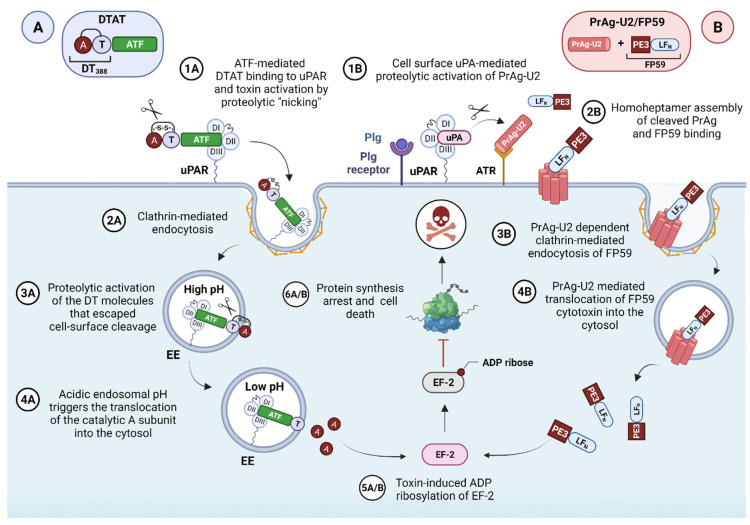
Putative mechanisms of action of ATF-fusions toxins: DTAT and uPA-activated PrAg-U2/FP59. (A) The uPAR-targeted cytotoxin DTAT consists of the 388-amino acid portion of DTAT, comprising the translocation (T) and N-terminal catalytic A domains of DT, fused to the amino-terminal fragment of uPA; (1A) The ATF targeting moiety mediates DTAT delivery and binding to the target uPAR-expressing cells. Cell-surface furin protease or furin-like proteases activate DT by proteolytic “nicking” of the arginine-rich bridge connecting the A and T domains, which remain linked by a disulfide bond; (2A) DTAT is then internalized in clathrin-coated pits into early endosomal vesicles (EE), where (3A) proteolytic activation of toxin molecules that escaped cleavage by cell-surface proteases may occur. (4A) Upon endosome acidification, the T domain undergoes a conformational change, inserts into the endosomal membrane, and forms a pore through which the catalytic domain A translocates to the cytoplasm, after concomitant reduction of the interdomain bridging disulfide bond. (5A) Here, the A domain inactivates the translation elongation factor 2 (eEF-2) by ADP-ribosylation, causing irreversible inhibition of protein synthesis and subsequent cell death, via apoptosis. A single molecule of toxin delivered to the cytosol is sufficient to kill the target cell [172,173,179,211]. (B) PrAg-U2/FG59 is a dual-component prodrug designed to be selectively activated by uPA on the surface of uPAR-overexpressing tumor cells. (1B) In the presence of pro-uPA and plg, receptor-bound PrAg-U2 undergoes a uPA-mediated activating cleavage and, subsequently, (2B) self-associates into a barrel-shaped heptamer, which binds the recombinant cytotoxin FP59. (3B) The resulting toxin complex is then internalized into endosomes where FP59 translocates to the cytosol in a PrAg-dependent manner (4B). (5B, 6B) As for DTAT, cell death occurs via apoptosis following protein synthesis arrest induced by EF2 inactivation catalyzed by the ADP-ribosylating domain of *Pseudomonas* exotoxin A (PE3) included in the fusion protein FP59 [83,212]. Abbreviations: ATR, anthrax toxin receptor. Created with BioRender.com.

In this context, most of the published work has been performed by the group of Liu, Leppla, and Bugge, who elegantly pioneered this field with the construction of an engineered anthrax toxin protective antigen (PrAg)-based prodrug named PrAg-U2 (Table 3). This was obtained by replacing the native furin cleavage site in PrAg, ^164^RKKR^167^, with a urokinase activable sequence ^163^PGSGRSA^169^, called U2. PrAg-U2 was then combined with a PrAg-dependent fusion protein, FP59, consisting of anthrax toxin lethal factor residues 1–254 (containing the N-terminal PrAg binding domain required for LF internalization) fused to the catalytic domain of *Pseudomonas* exotoxin A (PE3), to improve its cytocidal efficacy [82,83,209,210]. A comparison between the putative mechanism of actions of PrAg-U2/FP59, as a uPA-activated prodrug, and one of the previously described uPAR-targeted cytotoxins, DTAT, is illustrated in Figure 7.

The tumoricidal potential of the dual prodrug, PrAg-U2/FP59, has been successfully verified both in vitro on several malignant urokinase and anthrax toxin receptors expressing cell lines (nanomolar to picomolar IC_50_ values) [83,209,213], and in vivo, both in murine syngeneic and human xenograft tumor models of diverse origin, where significant anti-tumor effects were observed following local and systemic PrAg-U2/FP59 administration [209,214,215]. A dose–response relationship with both antitumor efficacy and systemic toxicity was observed, the latter being prevented upon concurrent treatment with the anti-inflammatory dexamethasone [209,214,215]. IHC analysis following maximum tolerated dose (MTD) assessment confirmed that, apart from endowing the engineered toxin with potent tumor-selective cytotoxicity, the switch from furin to urokinase activation noticeably attenuated or completely abolished toxicity to major organ systems, even at high toxin dosage, compared to the native PrAg [82,209]. Remarkably, total tumor regression and 30% complete histologic remission were reported in subcutaneous NSCLC bearing athymic nude mice after systemic administration of tolerated doses of PrAg-U2/FP59 [214]. The absolute requirement for the functional assembly of an active uPA/uPAR cell surface template for uPA-mediated plasminogen and subsequent PrAg-U2 activation in vivo was impeccably demonstrated by the complete lack of treatment response in tumor-bearing plg, uPA, and uPAR single-deficient mice challenged with the engineered toxin, as opposed to wild-type mice, which, in contrast, became terminally ill [82,83,209]. Similarly, deficiency of the cognate uPA inhibitor PAI-1 lowered the IC_50_ of this chimeric toxin [82,209]. As mentioned above, this study provided unequivocal evidence for the critical role of uPAR in the generation of cell-surface uPA activity in vivo.

After this pioneering work, additional improvements have been attained by the authors to further increase the tumor selectivity of the engineered anthrax toxins. This objective was achieved by combining PrAg protoxins with different specificity determinants, respectively, the formerly described PrAg-U2 and the MMP-activated PrAg protein PrAg-L1 [216] (Table 3). An intermolecular complementation approach was devised to target tumor cells displaying high levels of both proteolytic activities on their cell surface. As the LF binding site spans two adjacent monomers of the PrAg oligomeric heptamer, the two uPA- and MMP-activated PrAg protoxins were further mutated in one of the LF binding subsites so that efficient intracellular translocation would only occur upon intermolecular assembly of a hetero-heptameric pore, consisting of both uPA- and MMP-derived PrAg mutated monomers forming a functional LF-binding site [216]. The combination of uPA-activated PrAg-U2-R200A and MMP-activated PrAg-L1-I210A variants proved to be the most effective in vitro.

In vivo, when administered in combination with either FP59 or LF, the inter-complementing toxin PrAg-U2-R200A/PrAg-L1-I210A showed effective anti-tumor activity toward various types of aggressive transplanted tumors compared to the individual PrAg variants, signifying intermolecular complementation as the key mechanism to achieve potent tumoricidal efficacy [216,217]. In an anti-tumor potency comparing assay, PrAg-U2-R200A/PrAg-L1-I210A was at least as effective as an equivalent dose of PrAg-U2. However, the maximum tolerated dose of PrAg-U2-R200A/PrAg-L1-I210A was higher than that of PrAg-U2, evidence of the higher tumor specificity achieved by the dual-specific complementing mixture. This was further confirmed by the marked decreased toxicity of PrAg-U2-R200A/PrAg-L1-I210A compared to the parental PrAg-U2 and PrAg-L1 [216]. The present study provides POC for the rational design of engineered toxins with exceptional specificity for selected tumor types designated with unique combinations of overexpressed pericellular proteases not found on normal tissues. The efficacy of this reengineered anthrax toxin was recently demonstrated in a small clinical study on canine oral mucosal melanomas (OMM) with all five dogs showing stable disease after intratumoral treatment and no outward signs of systemic toxicity [218]. However, survival after treatment, and potential toxin-associated immunogenicity, were not assessed in these reports and require further research to clinically advance this promising fusion toxin.

The protease-activated prodrug approach has been successfully applied by the same authors to optimize the therapeutic index of DT-fusion toxins such as DT388GMCSF. This monospecific chimera combines the catalytic and translocation domains of DT (DT388, as for DTAT) with the granulocyte-macrophage colony-stimulating factor (GMCSF), which stimulates acute myeloblastic leukemia (AML) blast cells’ growth [219]. DT388GMCSF belongs to the first generation of DT chimeric toxins for refractory and relapsed AML comprising DTIL3 and DTAT [177,181,209]. Despite the promising preclinical and clinical toxicity exhibited by DT388GMCSF, its clinical applicability was limited by the pronounced hepatotoxicity due to the non-specific targeting of normal liver GM-CSF receptor (GM-CSFR)-expressing cells, such as macrophages [209,220]. Taking advantage of uPA/uPAR overexpression in AML relative to normal tissues [213,221], coupled with the DT-dependency on cell-surface proteolytic activation, DT388GMCSF’s specificity to AML was enhanced by engineering the DT furin site to acquire an AML-selective protease system, such as uPA/uPAR, for PrAg-U2. The uPA-cleavable sequence (U2) insertion yielded DTU2GMCSF (Table 3). Preclinically, this dual-specificity fusion toxin exhibited high toxicity on different leukemic cell lines with picomolar IC_50_ values. The cytotoxic effect proved to correlate to the cell surface density of both receptors and total uPA levels, as also shown by the reduced toxicity or resistance seen in normal cells expressing uPAR or GMCSF-R alone, as well as in the sensitive cells following pretreatment with receptor-blocking antibodies [209]. Interestingly, compared to the former studies, the minimal receptor expression levels needed for DTU2GMCSF antitumor activity were determined and found to be 236 and 54 receptors/cell for GM-CSFR and uPAR, respectively. These results suggest that there may be a threshold effect for uPAR expression that may mediate drug effect similar to what has been found for other cell-surface tumor targets such as HER2 and c-MET [222]. Although preclinical evaluation of DTU2GMCSF has not yet been performed in vivo, this study provides a POC for the efficacy and increased specificity of bispecific fusion protoxins. From a broader perspective, the combination of targeted delivery with protease-mediated toxin activation strategies, accomplished with DTU2GMCSF, may provide an optimum approach to improve the therapeutic index of both bi- and monospecific fusion toxins aimed for cancer treatment, and in the case of DTU2GMCSF, for potential AML therapy, and therefore merits further investigation.

In this context, an additional example is provided by ALA, a novel uPAR-targeted recombinant protoxin, recently developed using the scorpion toxin peptide AGAP, which has been recognized as a new promising anti-cancer drug candidate [223] (Table 3). ALA design combines the strategies of both classes of ATF-fusion toxins as it involves ATF fusion to the AGAP domain along with two uPA-cleavable sites. The conjugation to ATF improves AGAP suitability as a putative therapeutic peptide, a goal generally challenged by the intrinsic biologic properties of natural peptides including, among others, fast clearance, short half-life, limited permeability and stability, and accumulation in non-targeted healthy organs [224]. The targeting and cytotoxic potential of ALA was evaluated in vitro with high uPAR-expressing breast cancer cell lines, including MDA-MB-231, and uPAR-negative control cells. ALA was selectively delivered to and inhibited the proliferation of the target cancer cells in a dose- and receptor-dependent manner compared to the control cells lacking both uPAR and uPA required for the toxin proteolytic activation [223]. AGAP-induced cell apoptosis may underlie ALA’s cytotoxic mechanism of action as suggested by flow cytometry and western blot analysis [223]. In addition to confirming ATF as an effective targeting vehicle, this preliminary study highlights the potential of ALA as a novel antitumor candidate for treating uPAR-positive malignant tumors.

Collectively, these findings motivate further pre-clinical and clinical evaluation of uPAR-targeted fusion toxins, alone or in combination with conventional treatment modalities. Toxin-induced immunogenicity will most likely remain the main hurdle challenging their long-term therapeutic application in immunocompetent patients, as seen for other IT/LT. However, the implementation of strategies to mitigate or neutralize host anti-drug antibody (ADA) responses, as successfully achieved with eBAT, renews hopes to cope with or potentially overcome this problem.

**Table 3:** Mono- and bi-specific ATF-fusion recombinant protoxins targeting the urokinase receptor in cancer.

LT Name	Toxin	Origin of the Toxin	Additional Surface Target	Application	Model System	Ref.
PrAg-U2/FP59	AT	*Bacillus anthracis*	/	Preclinical	Human and mice uPAR-overexpressing malignant cell lines; syngeneic mouse and human xenograft cancer models of diverse origin; Pilot POC study of canine OMM	[82,83,213,214,215,217,218]
PrAg-U2-R200A/PrAg-L1-I210A	LF/FP59	*Bacillus anthracis*	/	Preclinical	Syngeneic mouse models of diverse origin	[216]
DT388GMSF	DT	*Corynebacterium diphtheriae*	GMCSFR	Preclinical	Human AML cell lines	[219]
ALA	AGAP	*B. martensii Karsch*	/	Preclinical	Human breast cancer cells	[223]

Abbreviations: PrAg, protective antigen; AT, anthrax toxin; DT388, diphtheria toxin (truncated form); GMSF, granulocyte-macrophage colony-stimulating factor; POC, proof-of-concept; OMM, oral mucosal melanomas; GMCSFR, granulocyte-macrophage colony-stimulating factor receptor.

#### 3.2.3. uPAR: A Novel Molecular Target for Antibody-Drug Conjugates (ADCs)

Conceptually similar to ligand-based toxin conjugates, ADCs represents an emerging and actively growing class of anti-cancer therapeutics that has reached clinical and regulatory milestones, with a total of 11 FDA-approved ADCs currently marketed for oncologic indications, over hundreds under advanced clinical evaluation, and many more in preclinical development. They provide another example of cancer-specific prodrugs. The toxin moiety is covalently linked to a tumor-targeting antibody via specialized serum-stable chemical linkers that, after target receptor-mediated internalization, ensure the cargo release and processing selectively into the desired cancer (or stromal) cells, leading to their targeted eradication [225,226,227].

**Figure 8:**
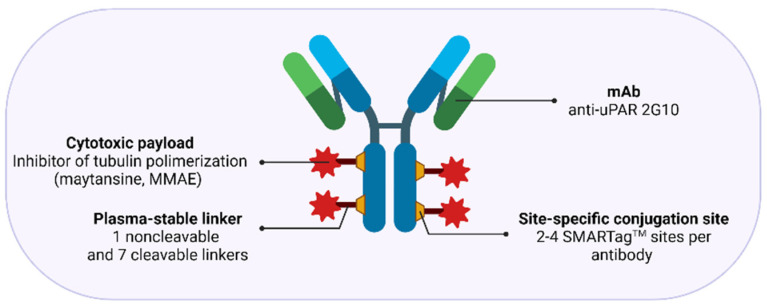
Design of anti-uPAR 2G10 ADCs. The parental anti-uPAR Ab 2G10 was site-specifically modified via the SMARTag^®^ technology, which genetically incorporates an aldehyde tag to the designated location within the antibody, yielding homogeneous ADC with defined attachment sites for the payload. One or two aldehyde tags were incorporated into the IgG heavy chain (Fc portion), resulting in two or four conjugation sites. Inhibitors of tubulin polymerization (maytansine and MMAE) covalently linked to Hydrazino-iso-Pictet-Spengler (HIPS)-functionalized cleavable or non-cleavable linkers were then conjugated to the reactive 2G10 Fc sites to generate nine site-specifically conjugated 2G10 ADCs. Created with BioRender.com.

This approach is now stepping into the uPAR field, with only one relevant example recently reported by the group of Craik et al., using the 2G10 antibody (introduced in Section 3.1 “uPAR-targeted radionuclide therapy”). Based on the encouraging preclinical results obtained with both naked Ab and the ^177^Lu radiolabeled version and the practical limitations inherent in the use of radioimmunoconjugates [166,167], a panel of nine 2G10 ADCs were designed and produced bearing the antimitotic tubulin inhibitor payloads, maytansine, and monomethyl auristatin E (MMAE), site-specifically conjugated via different linkers [228] (Figure 8).

Besides being a high-affinity binder to uPAR, 2G10 displays fundamental properties that render it an excellent Ab scaffold for ADCs, namely slow dissociation rate constant (k_off_) and capability to induce receptor-mediated internalization. The nine ADCs were tested and validated in vitro with the reference TNBC cell line, MDA-MB-231, and the cell-derived xenograft mouse model [228]. All ADCs were efficiently internalized and, at the highest concentration, induced a ~17–60% reduction in cell viability relative to the vehicle-treated cells [228]. In vivo, the anti-uPAR ADC that exhibited superior efficacy, leading to a one-third decrease in tumor volume, comprised an MMAE payload with a cathepsin B cleavable linker, named 2G10-RED-244-MMAE. Interestingly, the maximal dose used, 10 mg/kg, was a third lower than that of naked 2G10 causing tumor regression in the same TNBC xenograft model [167].

At this dosage, there was no evidence of toxicity based on body weight determination or clinical observations. However, as already noticed, the use of this antibody, specific to human uPAR, in a xenograft mouse model excluded the evaluation of drug-related toxicity to normal mouse cells along with ADCs effect on the mouse stroma, thus demanding an in-depth investigation.

In addition to validating uPAR as a novel potential target for ADC-based constructs, this study supports the future development of 2G10-RED-244-MMAE as a promising anti-uPAR ADC candidate.

Given the availability of well-characterized anti-uPAR monoclonal antibodies, and the expanding clinical success of ADCs for cancer treatment, the development of ADCs targeting uPAR is expected to open new therapeutic avenues for aggressive cancers, such as TBNC, where there is a paucity of molecular targets.

### 3.3. uPAR-Targeted Nanoparticles as Potential Anti-Tumor Theranostic Platforms

uPAR-targeting moieties, such as the AE105 peptide and ATF, have been successfully employed by several groups to engineer and guide drug-loaded nanoparticles (NPs) selectively to tumor sites (Table 4).

In addition to the improved site-specific drug delivery and reduced toxicity conferred by the active tumor targeting, nanoscale carrier systems inherently display fundamental advantages over conventional chemotherapy and are, therefore, gaining increasing attention in cancer research and treatment [229]. Beyond their favorable pharmacokinetics (e.g., prolonged blood circulation half-life), they provide versatile platforms that can be functionalized with multiple agents and, thus, enable different biomedical applications, including imaging and diagnosis, besides drug delivery [229]. These unique properties have fostered the development of tumor-targeted theranostic nanoparticles to achieve efficient tumor-selective imaging and therapy [230]. Magnetic iron oxide nanoparticles (IONPs) have emerged as excellent candidates for this purpose due to their intrinsic properties as clinically valuable MRI contrast agents and versatile surface chemistry that allows incorporating various imaging modalities [229,230,231]. Their biological safety in humans has been assessed, with non-targeted IONPs currently in use or under clinical development to detect liver tumor lesions or lymph node metastases [232,233,234].

Yang et al., were the first to develop multifunctional uPAR-targeted IONPs [235]. The ATF peptide was conjugated to amphiphilic polymer-coated IONPs carrying conventional chemotherapeutic drugs, such as doxorubicin (Dox), gemcitabine (Gem), or cisplatin (Cis) [236,237,238,239]. The encapsulated payloads were effectively internalized and released into uPAR-positive breast and pancreatic cancer cells, following receptor-mediated endocytosis, and produced significant cytotoxic effects compared to equivalent concentrations of free drugs and non-targeted drug-loaded IONPs. A low level of non-specific uptake was also detected for the latter upon incubation for a longer time, consistent with previous observations [236,237,238]. Importantly, the drug-release process of all the investigated payloads was pH-dependent, and the release rate increased with decreasing pH values. This pH-sensitive behavior is highly desirable for targeted cancer therapy as it improves drug-delivery efficiency at the tumor site and prevents premature drug release into the bloodstream (pH 7) by exploiting the unique characteristics of tumors, such as the slightly acidic pH, besides cancer cell lysosomes [240]. In the case of the Gem-loaded IONPs, using a pH-sensitive lysosomally cleavable linker peptide for conjugating the chemotherapeutic Gem on the polymer surface of the IONPs achieves Cathepsin B-dependent drug release into intracellular lysosomes.

In vivo studies were conducted on mice bearing human orthotopic breast or pancreatic tumor xenografts [236,238,239]. Interestingly, a mixture of human and mouse ATF was insightfully employed to target both human tumor cells and mouse-derived tumor stromal cells in the mouse models used, unlike the previously reported studies [236,238]. For NIR optical imaging, the ATF moiety was labeled with NIR dyes such as NIR-830 or Cy5.5. This potential multimodal imaging approach provides a valuable tool to validate and track the IONPs and, therefore, better investigate tumor targeting and biodistribution of nanoconstructs in animal models. Following systemic delivery, selective accumulation of the drug-loaded ATF-IONPs was observed in both tumor and stromal cells, especially at the tumor margin, congruent with the uPAR expression profile [236,238]. As expected for systemically delivered IONPs, accumulation in normal organs was mostly found in the liver and spleen, due to the non-specific uptake of IONPs by resident macrophages (reticuloendothelial system, RES), but not in other organs (heart, liver, intestine, and kidney) or tissues surrounding the tumor lesions.

The possibility to specifically target both tumor and stromal compartments, as in the present example, provides a double benefit: it allows overcoming or weakening the dense stromal barrier that severely hampers tumor perfusion by therapeutics and support tumor progression; at the same time, it increases the intratumoral drug delivery and retention of the targeted nanoparticles and thus the sensitivity of tumor detection through imaging. This is particularly relevant when targeting uPAR positive tumors where the stromal compartment primarily accounts for the prominent uPAR expression, as detailed in the previous sections. Due to the high level of IONPs delivered into the tumor areas, significant tumor growth inhibition was observed in both tumor models, with no apparent systemic toxicity compared to free drugs, such as Dox. As anticipated, highly proliferative cancer cells were more sensitive to Dox than the slowly dividing stromal cells, as shown by the higher number of IONPs positive cells found in the tumor stroma after the treatment [238]. This observation underscores the importance of tuning the targeting strategy and thus selecting the optimal payload to ensure the efficient killing of the desired cell types without or marginally affecting normal healthy cells. Moreover, the presence of drug-resistant residual tumors could also be identified non-invasively by NIR or MRI, signifying the value of uPAR-targeted theranostic agents, such as nanoparticles, for imaging-guided therapy.

**Figure 9:**
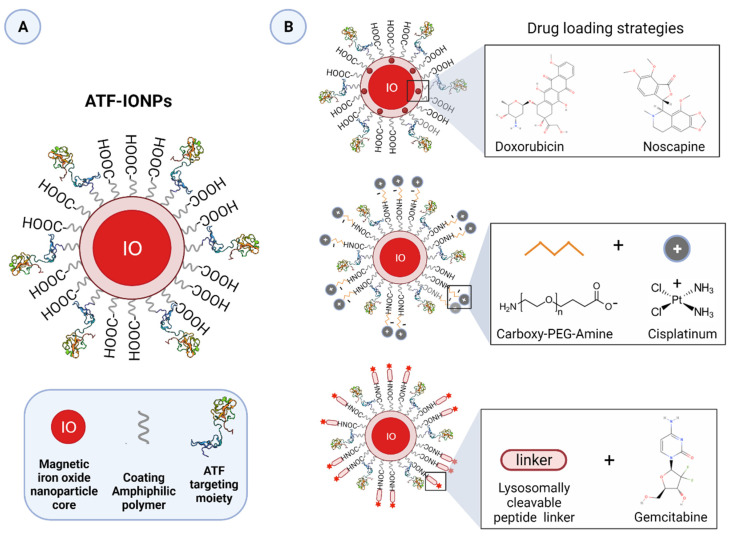
Composition and drug loading strategies of uPAR-targeted IONPs. (**A**) Most of the uPAR-targeted NPs developed up to date consist of magnetic IONPs coated with amphiphilic polymers. In addition to high stability, these polymers provide active functional carboxyl groups (COOH) on the particle surface for bioconjugation of targeting moieties, such as recombinant ATF (PDB accession number: 1URK) or other uPAR-peptide antagonists (not shown in the figure). (**B**) Different drug encapsulation methods can be utilized based on the physicochemical properties of the cargo, as well as drug-delivery strategies. Direct adsorption of the drug on the NP surface through non-covalent interaction increases the amount of drug encapsulated and efficiency of release into the targeted cells, compared to covalent linking [242]. Using this direct encapsulation method, doxorubicin and noscapine, two hydrophobic anticancer drugs, were physically adsorbed onto the amphiphilic polymer coating of ATF-IONPs via hydrophobic interactions (top) [236,237]. Similarly, the chemotherapeutic cisplatin (middle) was conjugated on the surface of ATF-PEG-IONPs via a coordinate bond between the positive charge of platinum (Pt^+^) with the negative charge of the coating polymer’s carboxylate groups (O=C-O-, Lewis base) [239]. In both cases, drug release is triggered at acidic pH values, typical of the hypoxic tumor milieu or intracellular lysosomes. Alternatively, more controlled drug release may be achieved via linker-mediated adsorption [242]. Using a pH-sensitive lysosomally cleavable peptide linker, gemcitabine, a hydrophilic drug, was covalently conjugated on the polymer surface of ATF-IONPs, achieving cathepsin B-mediated intracellular drug release (bottom) [236]. Created with BioRender.com.

**Figure 10:**
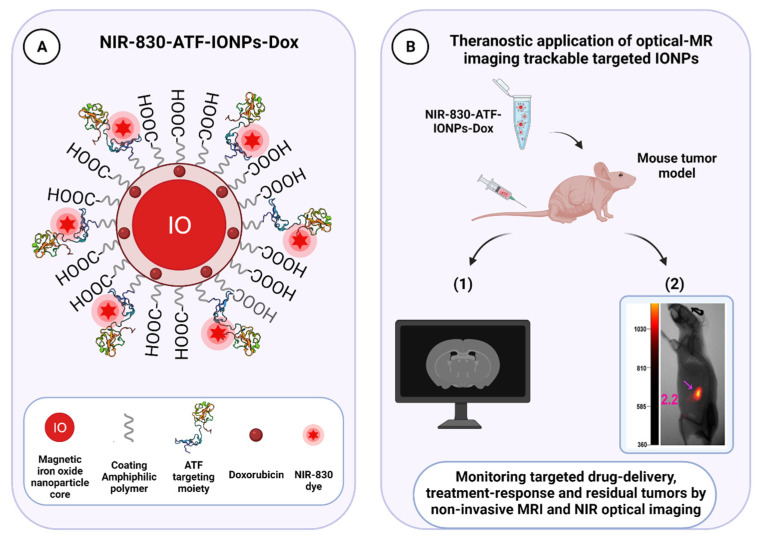
Theranostic application of uPAR-targeted IONPs. (**A**) Schematic representation of optical-MRI imaging trackable Dox-loaded ATF-IONPs [238,239]. The near infrared (NIR)-830 dye was conjugated to the targeting moiety, ATF (PDB accession number: 1URK), for optical imaging. Incorporating multiple imaging modalities on the same nanocarrier system provides a valuable way to validate and track the IONPs and, therefore, better investigate tumor targeting and biodistribution of nanoconstructs in animal models. (**B**) In vivo, these theranostic-targeted nanoparticles allowed for real-time and non-invasive MRI and NIR-optical imaging assessment of targeted drug delivery and distribution, tumor response to treatment, and detection of small drug-resistant residual tumors in orthotopic human pancreatic (shown in 2, reproduced from [238] under the Creative Commons Attribution (CC BY-NC) License) and breast cancer xenograft models, thus supporting the translation potential of these agents for image-guided therapy of cancer. Created with BioRender.com.

Using the same approach, Abdalla et al., have developed optical-MR imaging trackable, pH-responsive ATF-IONPs for the selective delivery of noscapine (Nos), a tubulin-binding anti-cancer agent, to prostate cancer cells [241]. The Nos-loaded targeted IONPs markedly enhanced intracellular Nos accumulation, leading to a ~6-fold increase in cell death compared to the free, untargeted drug. Although preclinical evaluation of the following NPs has not yet been reported, this theranostic approach holds potential for the management of prostate cancer patients. It further underscores the utility of targeted delivery and drug encapsulation to improve the therapeutic index and pharmacokinetics of anticancer drugs, such as noscapine or doxorubicin [241]. The following figures schematically illustrate the composition, drug loading strategies (Figure 9), and theranostic applications (Figure 10) of some of the above-described uPAR-targeted nanoparticles.

Dual-targeted strategies based on the simultaneous targeting of two tumor-selective targets or combinatorial antitumor therapy offer another appealing way to synergistically amplify the therapeutic response while minimizing the occurrence of drug resistance. In this context, Ahmed et al., recently reported, for the first time, the development of MRI-trackable dual-targeted IONPs to selectively and preferentially deliver the chemotherapeutic paclitaxel (PTX) to prostate cancer cells, taking advantage of the simultaneous overexpression of uPAR and the luteinizing hormone-releasing hormone receptor (LHRH-R) in this cancer type [243]. The IONPs were conjugated to the receptor’s targeting peptides, AE105 for uPAR, and a modified LHRH peptide for the LHRH-R. These LHRH-AE105-IONPs were efficiently internalized and accumulated in the treated cancer cells compared to normal prostate epithelial cells, as visualized by MRI imaging [243]. Accordingly, LHRH-AE105-IONPs-PTX showed enhanced cell killing capability versus the single-receptor-targeting IONPs-PTX (2-fold), non-targeted IONPs-PTX, and free PTX (3-fold). Moreover, the concentration of free PTX required to attain similar cytotoxicity as LHRH-AE105-IONPs-PTX IONP was 10 times higher [243]. These findings further confirm the significance of dual-targeted delivery as a powerful method to improve standard drug dosage and thus the quality of life of cancer patients.

To overcome the challenges associated with Dox chemotherapy, uPAR-targeted dual drugs co-encapsulated nanoparticles (NPs), carrying Dox and curcumin (CUR), were constructed to treat lung cancer, using the uPAR-targeting peptide U11 [244,245]. The in vitro and in vivo antitumor effects of these U11-DOX/CUR NPs ware evaluated in DOX-resistant human lung cancer cells (A549/ADR) and corresponding cell line-derived xenograft mouse models. Both U11-DOX/CUR- and untargeted-NPs had more synergistic cytotoxicity in vitro, enhanced tumor distribution, anticancer efficacy, and tolerability in vivo than the free drugs alone and their combination [244], suggesting the efficiency of NP-mediated drug delivery. However, the U11-DOX/CUR-NPs exhibited higher intratumoral accumulation (at 72 h post-injection) and cytotoxicity than the non-U11 decorated counterparts and single-drug loaded NPs. Combined, the results from these studies underline that the targeting effect of uPAR can significantly affect the NP distribution by increasing intracellular and thus total tumor uptake to a greater extent than the sole passive delivery due to the enhanced permeability and retention effect (EPR) effect. The present work also suggests that combination therapy via targeted nanocarriers may provide a superior therapeutic outcome to the current drug cocktail therapies.

Importantly, the use of uPAR-binding peptides as targeting moieties may prevent some of the shortcomings associated with ATF (e.g., undesirable agonist effects on vitronectin mediated cell adhesion and lamellipodia formation, as well as inherent protein-related drawbacks), as also suggested by Hansen et al. [246]. Recently, AE147 peptide-conjugated PEGylated liposomes carrying docetaxel (DTX) and linked to the fluorescent dye chlorin e6 (Ce6) were developed as a theranostic nanoplatform to treat uPAR-overexpressing metastatic tumors, with promising results [247].

uPAR-targeted theranostic NPs have also been developed to selectively deliver phototherapeutic agents to tumor sites, with a few valuable examples reported in the literature [248] and reviewed [19]. In this context, a relevant example is provided by uPAR-targeted indocyanine green-modified gold nanoshells, which were designed as dual-imaging (computed tomography, CT, and NIR imaging) and photothermal agents for local treatment of deep-buried pancreatic tumors [249]. After a single treatment, these agents could effectively ablate orthotopic pancreatic tumors, inhibit metastasis, and prolong survival, with no apparent toxicity, in comparison to the clinical radiotherapy intervention, iodine-125 (125I) interstitial brachytherapy (IBT-125-I), thus exhibiting great translational potential for effective and safe therapy of unresectable and metastatic pancreatic cancer patients [249].

Overall, the present data, although circumstantial, emphasize the feasibility of developing multifunctional biocompatible uPAR-targeted nanoparticles as theranostic agents for combined diagnosis and image-guided therapy of uPAR-overexpressing primary and metastatic tumor lesions. Further studies on the biodistribution, pharmacokinetic/pharmacodynamic, and toxicity profile of these agents in vivo will provide essential information for their potential translation into the clinics.

**Table 4:** uPAR-targeted nanoparticles and theranostic approaches.

Nanoparticle Composition	Cytotoxic Payload	Drug Release Mechanism	Imaging Modalities	Application	Model System	Ref.
Amphiphilic polymer-coated ATF-PEG-IONPs	Dox	pH-sensitive	MRI	Preclinical	Human breast cancer cells	[237]
Amphiphilic polymer-coated NIR-830-ATF-PEG-IONPs	Dox	pH-sensitive	MRI and NIR	Preclinical	Orthotopic human breast and pancreatic xenograft mouse models	[238]
Amphiphilic polymer-coated ATF-IONPs	Gem	pH- and lysosomal enzyme-dependent	MRI	Preclinical	Human pancreatic cancer cells; orthotopic human pancreatic xenograft mouse model	[236]
Amphiphilic polymer-coated NIR-830-ATF-PEG-IONPs	Dox; Cis	pH-sensitive	MRI and NIR	Preclinical	Mouse pancreatic cancer cells; subcutaneous and orthotopic human pancreatic xenograft mouse models;	[239]
Amphiphilic polymer-coated Cy5.5-ATF-IONPs	Nos	pH-sensitive	MRI and NIR	Preclinical	Human prostate cancer cells	[241]
Amphiphilic polymer-coated LHRH-AE105-IONPs	PTX	pH-sensitive	MRI	Preclinical	Human prostate cancer cells	[243]
Dual drugs co-encapsulated U11-NPs	Dox and Cur	pH-sensitive	/	Preclinical	Human lung cancer cells; human lung xenograft mouse model	[219]
AE147-PEG-Lipo	DTX	n/s	FI	Preclinical	Human breast cancer cells; human breast xenograft mouse model	[247]

Abbreviations: PEG, polyethylene glycol; IONPs, iron oxide nanoparticles; MRI, magnetic resonance imaging; NIR, near infrared; LHRH, luteinizing hormone-releasing hormone receptor; Lipo, liposomes; FI, fluorescence imaging.

### 3.4. uPAR: A Novel Target for Cancer Immuno- and Virotherapy

Cancer immunotherapy has now decisively emerged as a novel pillar of cancer care, proving the power of engaging or modulating the immune system in the fight against cancer [250]. Besides therapeutic antibodies, such as ADCs, described before, immune-checkpoint blockade and adoptive cell therapy via CAR T-cells have been a breakthrough in the treatment of several malignant tumors, achieving notable clinical success in recent years [250].

**Figure 11:**
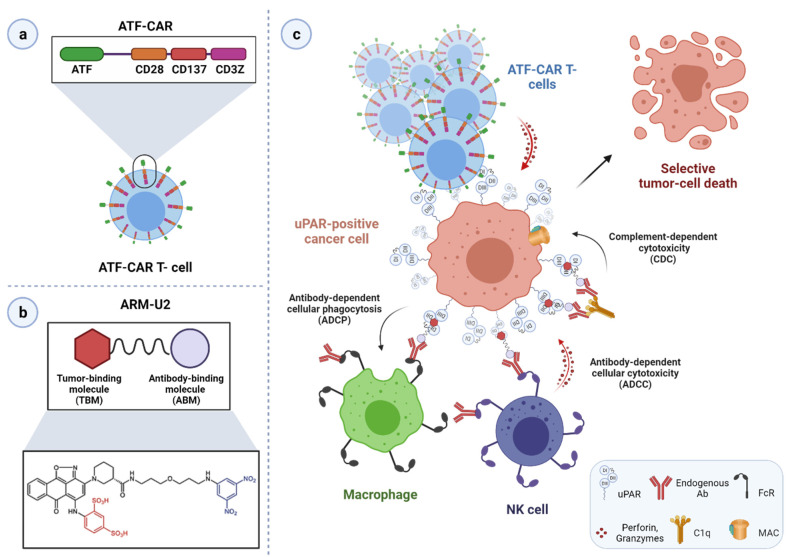
Schematic structure and putative mechanism of action of uPAR-directed immunotherapy approaches. (**a**) Wang et al. developed third-generation uPAR-targeted CAR T-cells using ATF as antigen recognition domain instead of the usual antibody-derived single-chain variable fragment (scFv) [251]. Besides the CD3 ζ-signaling domain, third-generation CARs incorporate endodomains from co-stimulatory molecules, in this case, CD28 and CD137 (also known as 4-1BB), to deliver the additional signals necessary to fully activate the T-cell, physiologically provided by the antigen-presenting cells (APC) [258]. (**b**) ARM-U2 is a uPAR-targeted antibody-recruiting small molecule comprising a derivative of the high-affinity uPAR inhibitor IPR-803 (bottom box), functioning as the target-binding molecule (TBM, shown in red), and a 2,4-dinitrophenol (DNP) moiety as the antibody-binding domain (ABM, depicted in blue). (**c**) Putative action mechanisms of ATF-CAR T cells (upper) and ARM-U2 (bottom). Upon uPAR-recognition and binding, mediated by the ATF domain, ATF CAR T-cells (upper) form a non-classical immune synapse (IS) with the target cancer cell, which mediates the T-cell cytolytic effector functions, including perforin and granzyme release, ultimately leading to selective tumor cell death [258]. The simultaneous binding of ARM-U2 to surface uPAR on the target cancer cell (by the TBM) and endogenous anti-DNP antibodies (recruited via the DNP moiety, ABM) results in the formation of a ternary complex, which can subsequently trigger selective immune-mediated cytotoxicity through different mechanisms. In the complement-mediated cytotoxicity, the C1q complement protein binds to the Fc part of the antibodies and activates the complement cascade, which culminates in the formation of the membrane attack complex (MAC) on the cancer cell surface and its lysis. Alternatively, the antibody’s Fc can also interact with the Fc-receptors expressed on the surface of various immune cells, such as macrophages or natural killer cells, followed by target cell phagocytosis (ADCP) or tumor-cell lysis via NK releasing potent oxidizing agents and protein toxins, such as granzyme and perforin (ADCC) [255]. Created with BioRender.com.

Wang et al. were the first to explore uPAR as a candidate target for CAR T-cells therapy in ovarian cancer [251] (Table 5). They constructed third-generation anti-uPAR CARs incorporating ATF as the antigen-binding domain in place of the usual antibody-derived single-chain variable fragment (scFv) to obtain shorter CAR frameworks with potentially reduced immunogenicity, a common hurdle for this line of therapy [252,253] (Figure 11a,c). The resulting ATF-CAR T-cells exhibited dose- and receptor-dependent specific cytotoxicity against the uPAR-positive cancer cells compared with the untransduced control T-cells (CT) [251]. Accordingly, cytokine and granzyme release was remarkably higher in the CAR T- versus the CT- treated group; no statistical difference was observed between CAR T- and CT-treated groups of receptor-deficient cells, thus confirming the specificity and efficiency of CAR T-cells. This promising report laid the groundwork for a subsequent study investigating the use of ATF-CAR T cells to treat senescence-associated disorders (including cancer) where uPAR is broadly expressed [254]. In vivo, treatment with such targeted CAR T-cells significantly improved the survival of mice harboring orthotopic lung adenocarcinomas and strikingly reduced liver fibrosis of different etiologies without eliciting detectable toxicity at the dosage used [254]. Collectively, these data provide proof of principle of a potentially broad therapeutic value of uPAR-targeted CAR T therapy in receptor-positive diseases, including various chronic pathologies, besides cancer. Future research will establish whether this approach has the required safety profile to be developed clinically as a cancer treatment or implemented in a combinatorial strategy.

uPAR targeting has also been explored for inducing selective immune-mediated clearance of uPAR-positive cancer cells via antibody-recruiting small molecules (ARMs) (Table 5). These synthetic agents consist of covalently linked bifunctional constructs comprising a tumor binding module (TBM), interacting with overexpressed cancer-specific biomarkers, and an antibody binding module (ABM), a hapten able to bind endogenous antibodies. By virtue of this design, ARMs have the ability to prime and direct downstream antibody-dependent effector mechanisms, including antibody-dependent cellular phagocytosis (ADCP) and cytotoxicity (ADCC), and complement-mediated cytotoxicity (CDP), toward the target-expressing cancer cells that are not efficiently recognized by the immune system on its own [255] (Figure 11b,c).

After a first generation ARM-U1 compound, containing uPA itself as the TBM [256], the authors synthesized a second-generation ARM, named ARM-U2, combining a restructured analog of IPR-803, a previously identified high-affinity uPAR inhibitor, as the TBM, and a 2,4-dinitrophenol (DNP) moiety as the ABM, to exploit the naturally occurring anti-DNP antibodies for cancer cell destruction [257]. In vitro, ARM-U2 induced concentration-dependent ADCP of uPAR-expressing A172 glioblastoma cells by IFN γ-activated U937 effector cells (E_max_ ~100 nM), as well as ADCC. In a subsequent in vivo study performed on a B16-uPAR mouse allograft model, ARM-U2 suppressed tumor progression, showing comparable effectiveness to the standard-of-care agent Dox, but without the substantial weight loss associated with this treatment, thus indicating an increase in selectivity and potentially improved side-effect profile, conferred by the active uPAR-targeting [257]. This work underscores the exciting promise of combining this class of small molecule immunotherapeutics with uPAR targeting to provide a potential novel treatment alternative to a broad range of uPAR-expressing deadly malignancies, which warrants further validation.

In addition to cancer immunotherapy, oncolytic virotherapy (OV) is another exciting fast-advancing field, which has exponentially gained interest over the last decade as evidenced by the growing number of viral platforms undergoing late-stage clinical trials and one FDA-approved OV in the USA [259]. The multifaceted mechanism of action of these agents, combining direct oncolysis and indirect activation of host anti-tumor immunity, can turn “cold” tumors (lacking tumor infiltrate) into “hot” by lifting the numerous immunosuppressive and physical barriers existing in the solid TME. The exceptional ability to modulate the TME renders OVs ideal combinatorial agents for improving tumor penetration and therapeutic outcome of immune and standard-of-care therapies in solid malignancies, as shown by the promising preclinical and clinical results attained by such combinatorial interventions [259,260]. In this context, the direct targeting of specific stromal components is one of the strategies being explored, with uPAR representing one of the emerging targets due to its abundant and ubiquitous expression in the tumor stroma of most cancer types [2].

Jing Y et al. were the first to investigate the feasibility of this approach. This group developed novel oncolytic measles viruses (MVs) fully retargeted against human (MV-h-uPA) or murine (MV-m-uPA) uPAR [261] (Table 5). The constructs were generated by displaying the ATF of human and murine uPA at the COOH terminus of a mutant MV-H glycoprotein (H_AALS_) that lacks the ability to bind its endogenous receptors, CD46 [261].

The availability of such species-specific platforms allowed the assessment of the OV therapeutic potential, the contribution of the stromal targeting to their anticancer activity, and, importantly, the relative pharmacokinetic/pharmacodynamic profile in immunocompetent syngeneic mouse models, which is typically an obstacle to the clinical development of oncolytic MVs as they cannot replicate in murine cells [261]. In vitro, MV-h-uPA and MV-m-uPA efficiently infected, replicated, and induced cytotoxicity in uPAR-expressing tumor and stromal cells, respectively, cancer-associated fibroblasts (CAFs) and endothelial cells, compared to the normal counterparts, in a receptor- and species-specific manner [261,262,263]. Successful species-specific fibroblast to tumor cell viral transfer was also observed [264]. Accordingly, murine CAFs infection by MV-m-uPA inhibited paracrine growth of co-cultured virus insensitive human cancer cells, which was instead stimulated by uninfected CAFs [262].

In vivo, systemic administration of both agents confirmed effective tumor targeting and led to significant cancer growth delay and prolonged survival in primary or metastatic, xenograft (MV-h-uPA), and syngeneic cancer models (MV-m-uPA), without any treatment-related toxicities or deaths [261,262,263]. The human xenograft mouse model used (where human cancer cells were MV-m-uPA resistant and MV-h-uPA sensitive, and vice versa, the murine stromal cells) consistently validated both MV-m-uPA direct stromal targeting and associated antitumoral effects observed in vitro [262]. Correlative studies investigating the underlying mechanisms showed that besides improved viral tumor entry and delivery to adjacent cancer cells, and increased tumor cell apoptosis, viral stromal targeting induced a significant modulation of in vivo tumor-stromal interactions. Notably, while treatment with MV-h-uPA showed superior tumor control effects to MV-m-uPA, the combination of both MV-h-uPA and MV-m-uPA outperformed monotherapy [262].

**Figure 12:**
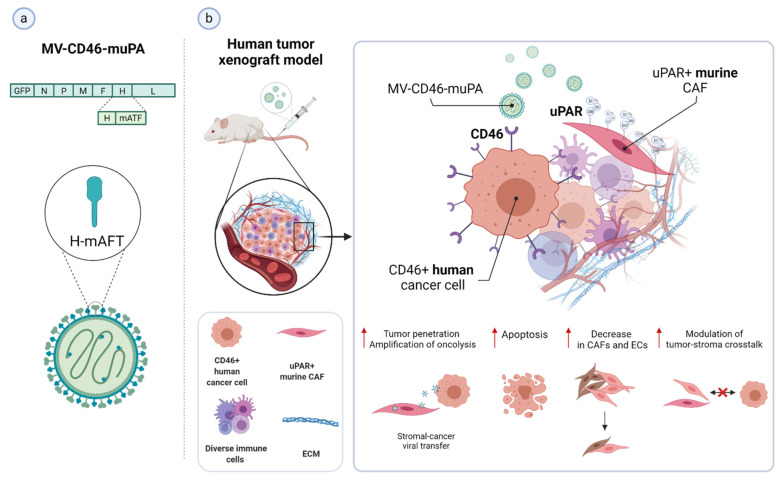
Schematic structure and in vivo antitumor effects of dual stromal and tumor-targeted oncolytic MV-CD46-muPA. (**a**) MV-CD46-muPA was generated by displaying the ATF of murine uPA as a C-terminal extension of unmodified MV-H glycoprotein (highlighted in the MV genome and structure) to allow simultaneous binding and infection of human cancer cells via the MV receptor CD46 and murine cells via murine uPAR. (**b**) The contribution of stromal and tumor targeting on MV’s overall anticancer efficacy in vivo was evaluated in human colon cancer xenograft mouse models, comprising CD46^+^ human cancer cells and uPAR^+^ murine stromal cells [264]. Systemically administered oncolytic MV-CD46-muPA led to improved antitumor effects and outcomes compared to vehicle or CD46 only targeted MVs. These effects resulted from the mouse uPAR-mediated stromal targeting ability of MV-CD46-muPA, lacking in the monospecific counterpart, and were associated with increased tumor viral penetration (via stromal-tumor viral transfer), apoptosis, decrease in murine stromal CAFs and endothelial cells (ECs), and viral-induced modulation of tumor-stroma interactions [264]. Created with BioRender.com.

Similar results were obtained in a recent follow-up study by the same group, where dual-targeted oncolytic MVs able to bind murine stromal (via murine uPAR) and human cancer (via CD46) cells were engineered and shown to successfully infect and lyse the target cells in a species-specific fashion, both in vitro and in vivo, in colon (HT-29) cancer xenografts, leading to improved tumor suppression and overall survival compared to vehicle CD46-only targeted MVs [264] (Table 5, Figure 12).

In addition to validating uPAR as a biologically relevant target for oncolytic therapy, and stromal targeting, these encouraging results signify the advantages of targeting the tumor stroma for therapeutic gain in combinatorial regimens and uPAR-targeted oncolytic virus as viable anti-tumor and stromal agents, thus supporting their further preclinical and clinical development. While novel uPAR-targeted OVs continue to be developed [264], open questions for future research remain, such as the OVs’ exact mechanism of action or effects on other relevant pro-cancer uPAR-positive stromal cells such as TAMs, one of the prominent tumor-promoting stromal cells. Elucidating these aspects will be essential to optimizing the therapeutic potential of these powerful agents, which may benefit future cancer patient management.

**Table 5:** uPAR-targeted immune- and virotherapy anti-cancer approaches.

Approach	Application	Model System	Ref.
ATF-CAR T cells	Preclinical	Ovarian cancer cells	[251]
ARM-U2	Preclinical	Glioblastoma cells; mouse melanoma allograft model	[257]
Oncolytic MV-m-uPA, MV-h-uPA	Preclinical	Murine and human colon and breast cancer cells, CAFs. HUVECs and murine EC; breast and colon cancer xenograft models	[261,262,263,265]
Dual-targeted oncolytic MV-CD46-muPA	Preclinical	CD46-positive colon tumor cells, murine uPAR-expressing CAFs; human colon tumor xenograft;	[264]

Abbreviations: CAR, chimeric antigen receptor; ARM, antibody-recruiting small molecule; MV, measles viruses; m, mouse; h, human; CAF, cancer associated fibroblasts; HUVECs, human umbilical vein endothelial cells; EC, endothelial cells.

## 4. Conclusions, Challenges, and Future Perspectives

Research performed over the past three decades has demonstrated the enormous potential of targeting uPAR for cancer treatment. Delivering a cytotoxic insult to uPAR-expressing cancer cells is becoming a viable option that may lead to a superior antitumor effect (tumor eradication) compared to the receptor function inhibition (slow tumor growth), the first avenue pursued in the field of uPAR targeting.

Although anti-uPAR therapeutic agents are yet to enter the clinics, uPAR offers multiple opportunities for targeted therapy that might be beneficial in cancer and, potentially, other human diseases, especially those with a paucity of molecular targets. However, the complexity of tumors renders it arduous to find a univocal solution to target all types of diseases involving uPAR as for most, if not all, cancer targets. Another existing challenge is designing and selecting appropriate preclinical animal models that account for the species specificity of the uPAR targeting agents, most of which recognize human but not mouse uPAR, thus complicating the interpretation of therapeutic efficacy and safety profile of the intervention strategies targeting this receptor. Indeed, the baseline expression of uPAR in healthy organs such as the lungs and kidney raises concerns about potential “on-target off-tumor” toxicity that may prevent the clinical application of such therapies. Strategies such as bispecific targeting of uPAR and other cancer targets or interaction partners or combinatorial therapy involving distinct anticancer approaches are increasingly being investigated and renew hopes to tackle these issues and optimize the therapeutic profile of uPAR-directed and targeted anticancer agents, in general. Accordingly, the potential of stromal targeting conferred by uPAR may further boost the antitumor activity in stroma-rich tumors. More exhaustive preclinical and, especially, clinical investigations in the future will establish the true efficacy of uPAR-targeted therapy and definitively validate the targetability of uPAR in human cancer patients. The availability of clinically tested uPAR-imaging agents will help speed up this process while favoring the implementation of theranostics that may improve and tailor the management of uPAR-positive cancer patients.

## Figures and Tables

**Table 2:** Mono- and bi-specific ATF-fusion recombinant toxins targeting the urokinase receptor in cancer.

LT Name	Toxin	Origin of the Toxin	Additional Surface Target	Application	Model System	Ref.
DTAT	DT	*Corynebacterium diphtheriae*	/	Preclinical	Human GBM and HUVECs cells; Human GBM SC and IC xenograft models	[179,182]
DTAT13	DT	*Corynebacterium diphtheriae*	IL-13Rα2	Preclinical	Human GBM and HUVECs cells; Human GBM SC and IC xenograft models	[181,183]
DTATEGF	DT	*Corynebacterium diphtheriae*	EGFR	Preclinical	Human NSCLC cells; Human metastatic NSCLC IC xenograft model	[187]
eBAT	PE38	*Pseudomonas aeruginosa*	/	Preclinical	Human GBM, HUVECs, HNSCC, breast, ovarian, sarcoma and pediatric sarcoma cell lines; GBM SC and IC xenograft models; Adaptive dose-finding, phase I–II clinical trial for canine HSA	[191,192,195,196,197,199]
ATF-SAP	Saporin	*Saponaria officinalis*	/	Preclinical	Human bladder and triple negative breast cancer cell lines; bladder cancer SC xenograft models	[203,204,206,207]

Abbreviations: GBM, glioblastoma multiforme; HUVECs, human umbilical vein endothelial cells; HNSCC, head and neck squamous cell carcinoma; HSA, hemangiosarcoma; SC, subcutaneous; IC, intracranial.

## Data Availability

Not applicable.
